# Systematic Analysis of Cotton Non-specific Lipid Transfer Protein Family Revealed a Special Group That Is Involved in Fiber Elongation

**DOI:** 10.3389/fpls.2018.01285

**Published:** 2018-09-19

**Authors:** Chengsheng Meng, Yuanyuan Yan, Zhengwen Liu, Liting Chen, Yan Zhang, Xiuxin Li, Liqiang Wu, Guiyin Zhang, Xingfen Wang, Zhiying Ma

**Affiliations:** North China Key Laboratory for Germplasm Resources of Education Ministry, Co-Innovation Center For Cotton Industry of Hebei Province, College of Agronomy, Hebei Agricultural University, Baoding, China

**Keywords:** cotton, nsLTP, type XI, expansion, fiber development, fiber evolution

## Abstract

Non-specific lipid transfer proteins (nsLTPs) had been previously isolated from cotton fiber but their functions were unclear so far. Bioinformatic analysis of the tetraploid cotton genome database identified 138 *nsLTP* genes, falling into the 11 groups as reported previously. Different from *Arabidopsis*, cacao, and other crops, cotton type XI genes were considerably expanded and diverged earlier on chromosome At11, Dt11, and Dt08. Corresponding to the type XI genes, the type XI proteins (GhLtpXIs) all contained an extra N-terminal cap resulting in larger molecular weight. The research revealed that the expression of type XI genes was dramatically increased in fibers of tetraploid cotton compared with the two diploid progenitors. High-level of *GhLtpXIs* expression was observed in long-fibered cotton cultivars during fiber elongation. Ectopic expression of *GhLtpXIs* in *Arabidopsis* significantly enhanced trichome length, suggesting that *GhLtpXIs* promoted fiber elongation. Overall, the findings of this research provide insights into phenotypic evolution of *Gossypium* species and regulatory mechanism of nsLTPs during fiber development.

**HIGHLIGHT**

A specific group, type XI nsLTPs, was identified with predominant expression in elongating fibers of *Gossypium hirsutum* based on evolutionary, transcriptional, and functional analyses.

## Introduction

Cotton is one of the important commercial fiber crops worldwide. Cotton fiber initiates from the outer epidermis of the ovules, followed by extensive cell elongation (about 1,000~3,000 times) and cell wall synthesis (Basra and Malik, 1984; Kim and Triplett, 2001). In *G. hirsutum*, lint fibers start to develop prior to or on the day of anthesis, and fuzz fibers develop a few days later (Joshi et al., 1967; Stewart, 1975). Fiber quantity and length, to a large extent, determine cotton yield and quality of the resulting spun thread. Long-chain fatty acid (LCFA) functions as a regulator of endogenous ethylene biosynthesis in cotton ovules, which can maximize the extensibility of cotton fibers (Qin et al., 2007). However, it is unclear how LCFA is transported and accumulated on the outer epidermis of ovules to regulate fiber development. The nsLTP is one of the well-known protein families with bound lipids (Boutrot et al., 2008) such as LCFA. Detailed studies of gene expression and function of nsLTPs are paramount for addressing the above question.

The nsLTPs are widely distributed in the plant kingdom and are capable to bind to acyl groups, various phospholipids and other fatty acid groups with a broad binding affinity (Ostergaard et al., 1993; Kader, 1996; Kragelund et al., 1997; Sodano et al., 1997; Charvolin et al., 1999; Han et al., 2001). It has been revealed that most nsLTPs have a small molecular weight of about 9 kDa in higher plants (Kader, 1996). All known plant nsLTPs contain an N-terminal signal peptide which is characterized by an eight cysteine motif (ECM) backbone forming four conserved disulfide bonds that stabilize a hydrophobic cavity to enclose the lipid to shield the hydrophobic portions of the lipid (Carvalho and Gomes, 2007; Boutrot et al., 2008; Wong et al., 2017). Plant nsLTPs are deemed to be responsible for the shuttling of lipids across cytoplasm and between membranes and regulate the beta-oxidation of fatty acids in glyoxysomes and the intracellular fatty acid pools (Kader, 1996; Cheng et al., 2004). Multiple physiological and biological functions of nsLTPs have been suggested, including membrane and liposome biogenesis (Pyee et al., 1994), somatic embryogenesis (Lee et al., 2009; Chae et al., 2010; Edstam and Edqvist, 2014), pollen development (Zhang et al., 2010; Chen et al., 2011), stress resistance (Zhang et al., 2016), defense (Molina et al., 1993; Schweiger et al., 2013), and signal transduction (Sarowar et al., 2009).

Cotton nsLTP genes have been isolated from fibers decades ago (Ma et al., 1995). *GH3, Ltp3, Ltp6*, and *GhLTPG1* are proved to be specifically expressed in fiber cells and *Ltp3* expression reaches to a maximum at the late fiber elongation stage (Ma et al., 1995; Han et al., 2013; Deng et al., 2016). Expression analysis showed that *GhLtp6, GhLtp7, GhLtp8*, and *GhLtp11* are highly expressed during fiber initiation (Han et al., 2013). Regarded as seed trichomes, fiber initiation, and elongation are regulated by MYB genes as leaf trichomes (Guan et al., 2008; Pu et al., 2008; Machado et al., 2009; Walford et al., 2011; Huang et al., 2013; Wang et al., 2013; Liu et al., 2015). And cotton *LTP3* was regulated by a MYB protein (Hsu et al., 2005). Thus it is supported that *nsLTPs* are involved in fiber development. However, the gene family members and the biological function during fiber development are largely unknown of these a few cotton *nsLTPs* cloned previously. The availability of genome sequence of *G. hirsutum* and insights into trichome developmental mechanism enable us to understand these scientific issues.

In *Arabidopsis* and rice, nsLTPs can be classified into several types (I, II, III, IV, V, VI, VII, VIII, IX, XI, and nsLTPy) based on the sequence similarity (Boutrot et al., 2008). Currently, 51 *nsLTP* genes have been identified in *Arabidopsis* (Boutrot et al., 2008; Li et al., 2014), 63 in rape and maize (Li et al., 2014; Wei and Zhong, 2014), 58 in sorghum (Wei and Zhong, 2014), and 52 in rice (Boutrot et al., 2008). In the present study, 138 *nsLTP* genes were identified in *G. hirsutum* that considerably expanded in *Gossypium* species during fiber evolution. Both transcriptional and functional analyses suggested an important role of *GhLtpXIs* in promoting fiber elongation.

## Materials and methods

### Identification and bioinformatics analyses of *nsLTP* genes

The genome sequences of *G. hirsutum* TM-1, *Gossypium arboreum, Gossypium raimondii, Arabidopsis thaliana, Brassica rapa, Theobroma cacao, Oryza sativa*, and *Vitis vinifera* were downloaded from CottonGen (https://www.cottongen.org), CottonFGD (https://cottonfgd.org), TAIR (http://www.arabidopsis.org), BRAD (http://brassicadb.org), Cacao Genome Database (https://www.cacaogenomedb.org), RAP (http://rapdb.dna.affrc.go.jp/), and Grape Genome Browser (http://www.genoscope.cns.fr/externe/GenomeBrowser/Vitis/) networks, respectively. The local genomes including coding sequences and protein sequences were constructed with the blast-2.2.9 program downloaded from the national center for biotechnology information (NCBI) (ftp://ftp.ncbi.nlm.nih.gov/blast/executables/release/2.2.9/blast-2.2.9-ia32-win32.exe). Candidate *nsLTP* genes were identified by BLASTP against the local databases using published nsLTP protein sequences of *Arabidopsis* and *Brassica* (Li et al., 2014) as queries with a cut-off value of e^−5^. The deduced protein sequences were analyzed for their signal peptides using SignalP 4.0 (http://www.cbs.dtu.dk/services/SignalP). Then the mature proteins with more than 120 amino acids were discarded, followed by analysis of the ECM domains with BioEdit. The structural domains were further analyzed with Batch Web CD-Search Tool (http://www.ncbi.nlm.nih.gov/Structure/cdd/wrpsb.cgi) and the members of nsLTPs were finally confirmed with HMMER. ProtParam (http://web.expasy.org/protparam/) was used to calculate the molecular weights and isoelectric points of GhLtps.

Multiple alignments of the nsLTP protein sequences of ECMs were performed using Clustal X version 2.0 program. Phylogenetic trees were constructed with the method of Maximum likelihood or Neighbor-Joining using MEGA 6.0 in pairwise complete deletion and Amino Acid P-distance model. For statistical reliability, bootstrap tests were carried out with 1,000 replicates.

The downloaded CDS and genomic sequences of *GhLtps* were used to construct gene structures on Gene Structure Display Server 2.0 (http://gsds.cbi.pku.edu.cn/). Mapinspect software was used to generate chromosome location image of each *GhLtp* according to their starting position on chromosomes. The segmental duplication was defined as the following: (1) the length of aligned sequences cover >80% of the longer gene; (2) the identity of the aligned regions >80%; (3) only one duplication event was counted for tightly linked genes (Kong et al., 2013; Wei et al., 2013; Liu et al., 2014). Tandem duplication was defined on the basis of the criteria that tandem duplicated genes are located within 15 predicted open reading frames or within 30 kb of each other (Shin and Bleecker, 2003; Wang et al., 2011). The non-synonymous to synonymous substitution ratio (Ka/Ks) for each duplicated *GhLtp* gene pairs was calculated using KaKs_Calculator 2.0 software.

### Expression analysis of cotton *nsLTPs*

The RPKM (Reads Per Kilobase of gene model per Million mapped reads) values of *GhLtps* were extracted from our RNA-seq data using ovules [0 and 5 days post anthesis (DPA)] and fibers (10–30 DPA) collected from *G. hirsutum* cultivars including HY405, CCRI8, and ND601 (Ma et al., 2018). Expression pattern analysis and differential expression analysis were subsequently applied with online tools Trend (http://www.omicshare.com/tools/Home/Soft/trend) and Diffanalysis (http://www.omicshare.com/tools/Home/Soft/diffanalysis), respectively. The volcano plot was generated with Excel (Microsoft, United States) to display differentially expressed *GhLtps*. Transcriptome data of genome-wide gene expression in different organs and cotton species was obtained from cottonFGD (file name: fpkm.Ghir.NAU.txt.gz) and the FPKM (Fragments Per Kilobase of exon per Million fragments mapped) values of cotton *nsLTPs* were extracted for comparison between different tissues or organs and different *Gossypium* species. The heatmaps were generated with HemI version 1.0.

### Plant materials

The cotton cultivars (HY405 and ND601) were grown in the growing season in the field in Baoding, Hebei (Latitude 38°48′N, 115°25′E). Cotton ovules (0 DPA) and fibers (5, 10, 15, and 20 DPA) were collected at 14:00–16:00 and fast frozen in liquid nitrogen for qPCR. The cotton cultivar TM-1 were grown at 28°C day/25°C night with 10 h light/14 h dark cycles in greenhouse of Hebei Agricultural University and ovules and fibers were collected at 10 DPA followed by fast frozen in liquid nitrogen for gene amplification.

*Arabidopsis* including Col wild-type (WT) plants and transgenic plants were grown at 22°C with 16 h light/8 h dark cycles in growth chamber at the North China Key Laboratory for Germplasm Resources of Education Ministry. Mature rosette leaves and cauline leaves were collected from flowering plants and frozen in liquid nitrogen before RNA extraction.

### RNA extraction and quantitative real-time PCR analysis

Frozen tissues were ground to a fine powder in liquid nitrogen and the total RNA was extracted with RN09-EASYspin Plant RNA purification kit (Aidlab). After quality and quantity analysis with gel electrophoresis and NanoDrop 2000, the first cDNA strand was synthesized using the PrimerScript^TM^ RT Master Mix (TaKaRa). Then quantitative real-time PCR was performed with Fast Super EvaGreen qPCR Master Mix (US EVERBRIGHT®INC) on an ABI 7500 Real-Time PCR machine. Three biological replicates of each sample and three technical replicates of each biological replicate were used. The relative expression level of the target gene was calculated with the difference between the cycle threshold (Ct) of the target gene and reference gene (ΔCt = Ct_targetgene_ − Ct_referencegene_) and corresponded to 2^−Δ*Ct*^. Primers used for qPCR were designed against gene specific sequences of CDS and listed in Supplemental Table S1.

### Generation of transgenic plants overexpressing *GhLtpXIs* in *arabidopsis*

The full-length of *GhLtpXIs* coding sequences were PCR-amplified from cotton cDNA (RNA was extracted from ovules and fibers of TM-1) with primers listed in Supplemental Table S1, and the resulting products were cloned into pGreen 0229 harboring 35S promoter (Yu et al., 2004). After verification by nucleotide sequencing, the resulting vectors were transformed into Agrobacterium followed by transformation with floral dip into *Arabidopsis* WT (Col) plants. The transgenic plants were selected by BASTA and the first three mature rosette leaves were cut from the petioles after bolting for discoloration with gradient ethanol followed by observation of trichomes under microscope with 20 × objective lens. The trichome length was measured using cellSens Standard (Olympus, Japan). Statistical analyses were performed using Excel (Microsoft, United States) with *T*-test (tail = 1, type = 3).

## Results

### Identification and classification of *nsLTP* genes in *G. hirsutum*

To identify the entire collection of putative *nsLTP* genes in the *G. hirsutum* genome, local BlastP searches were conducted using 53 and 63 nsLTP protein sequences of *Arabidopsis* and *B. rapa* as queries on the whole genome of *G. hirsutum*. A total of 219 *GhLtp* genes were obtained. Each of the deduced protein sequences was analyzed with SingalP, after which 18 proteins lacking N-terminal signal sequences (NSS) were omitted. Since low molecular weight is a common character of nsLTP proteins, 46 mature proteins whose peptide length was more than 120 amino acids were not taken into consideration. Then 16 proteins lacking the Cys residues were excluded following manually scanning for the presence of the eight essential Cys residues and 1 protein without the M residue (starting residue) was removed. The remaining protein sequences were detected for the LTPAAI_LTSS structure using the Batch Web CD-Search tool. Subsequently, the GhLtp candidates were further verified according to the HMMER method. Finally, 138 genes of *G. hirsutum* coding the nsLTP were confirmed (Table 1).

**Table 1:** Putative *nsLTP* genes identified in the genome of *G. hirsutum*.

**Name**	**Gene ID**	**Chromosome location**	**Strand**	**CDS Length** **(bp)**	**AA^a^**	**SP^b^**	**MP^c^** **(AA)**	**ECM^d^**	**MP** **(MW^e^)**	**MP** **(pI^f^)**
**Type I**										
*GhLtpI1*	GhAt02g0264	A02:3177157,3177498	–	342	113	23	90	C-X9-C-X13-CC19-C-X1C-X21-C-X13-C	6524.57	5.94
*GhLtpI2*	GhDt09g2050	D09:47878499,47878894	+	396	131	26	105	C-X9-C-X13-CC19-C-X1C-X22-C-X13-C	7947.31	8.51
*GhLtpI3*	GhAt09g2302	scaffold2284A09:62025,62414	+	390	129	26	103	C-X9-C-X13-CC19-C-X1C-X22-C-X13-C	7224.5	9.03
*GhLtpI4*	GhDt10g1143	D10:18523835,18524209	–	375	124	27	97	C-X9-C-X15-CC19-C-X1C-X22-C-X13-C	7947.31	8.51
*GhLtpI5*	GhAt10g2324	scaffold2673A10:15446,15820	+	375	124	27	97	C-X9-C-X15-CC19-C-X1C-X22-C-X13-C	7267.53	9.33
*GhLtpI6*	GhAt09g0865	A09:56336354,56336788	–	435	144	27	117	C-X9-C-X15-CC19-C-X1C-X22-C-X13-C	7208.51	9.44
*GhLtpI7*	GhAt09g0866	A09:56392915,56393396	–	363	120	27	93	C-X9-C-X15-CC19-C-X1C-X22-C-X13-C	7208.51	9.44
*GhLtpI8*	GhDt09g0890	D09:34044857,34045338	–	363	120	27	93	C-X9-C-X15-CC19-C-X1C-X22-C-X13-C	7947.31	8.51
*GhLtpI9*	GhDt13g2459	D13:60480760,60481188	–	429	142	30	112	C-X9-C-X13-CC19-C-X1C-X22-C-X3-C	8049.51	7.4
*GhLtpI10*	GhDt09g2051	D09:47884653,47885018	+	366	121	30	91	C-X9-C-X13-CC19-C-X1C-X24-C-X13-C	7947.31	8.51
*GhLtpI11*	GhAt09g2303	scaffold2284A09:68186,68653	+	372	123	30	93	C-X9-C-X13-CC19-C-X1C-X24-C-X13-C	7235.53	9.16
*GhLtpI12*	GhDt12g2101	D12:54017392,54017830	–	354	117	26	91	C-X9-C-X14-CC19-C-X1C-X21-C-X13-C	7974.42	8.84
*GhLtpI13*	GhAt12g1921	A12:81939867,81940280	–	414	137	27	110	C-X9-C-X14-CC19-C-X1C-X21-C-X13-C	7396.58	6.4
*GhLtpI14*	GhAt08g0297	A08:3429656,3430509	–	351	116	26	90	C-X9-C-X13-CC19-C-X1C-X22-C-X8-C	6961.12	9.01
*GhLtpI15*	GhDt08g0389	D08:3976879,3977232	–	354	117	26	91	C-X9-C-X13-CC19-C-X1C-X22-C-X14-C	7863.11	4.03
*GhLtpI16*	GhDt08g0388	D08:3972723,3973067	–	345	114	24	90	C-X9-C-X13-CC19-C-X1C-X22-C-X14-C	7821.35	8.71
*GhLtpI17*	GhDt07g1376	D07:22260738,22261166	+	429	142	27	115	C-X9-C-X16-CC19-C-X1C-X24-C-X15-C	7821.35	8.71
*GhLtpI18*	GhDt05g1065	D05:9034074,9034505	+	432	143	54	89	C-X9-C-X14-CC19-C-X1C-X21-C-X13-C	7755.04	9.18
*GhLtpI19*	GhAt05g3889	scaffold1236A05:86713,87168	+	351	116	25	91	C-X9-C-X14-CC19-C-X1C-X21-C-X13-C	6935.9	5.36
*GhLtpI20*	GhAt11g0687	A11:6714268,6714704	+	351	116	25	91	C-X9-C-X13-CC19-C-X1C-X22-C-X13-C	7353.55	6.4
*GhLtpI21*	GhDt11g0804	D11:6889195,6889539	+	345	114	25	89	C-X9-C-X13-CC19-C-X1C-X22-C-X13-C	7947.31	8.51
*GhLtpI22*	GhAt08g0541	A08:8492350,8492783	+	354	117	25	92	C-X9-C-X14-CC19-C-X1C-X22-C-X13-C	7003.12	9.01
*GhLtpI23*	GhDt08g0632	D08:7953474,7953821	+	348	115	25	90	C-X9-C-X14-CC19-C-X1C-X22-C-X13-C	7936.16	4.03
*GhLtpI24*	GhDt01g0179	D01:1466417,1466764	+	348	115	21	94	C-X9-C-X14-CC20-C-X1C-X23-C-X13-C	7475.76	8.69
*GhLtpI25*	GhAt01g0134	A01:1242782,1243129	+	348	115	21	94	C-X9-C-X14-CC20-C-X1C-X23-C-X13-C	6524.57	5.94
*GhLtpI26*	GhDt07g0758	D07:9275887,9276400	–	399	132	32	100	C-X9-C-X14-CC19-C-X1C-X21-C-X13-C	7794.32	8.71
*GhLtpI27*	GhDt12g2481	D12:57766428,57766799	+	372	123	23	100	C-X9-C-X16-CC19-C-X1C-X21-C-X13-C	7993.38	8.51
*GhLtpI28*	GhAt12g2346	A12:86018663,86019034	+	372	123	23	100	C-X9-C-X16-CC19-C-X1C-X21-C-X13-C	7449.68	8.69
**Type II**										
*GhLtpII1*	GhAt09g0486	A09:38767755,38768036	–	282	93	25	68	C-X7-C-X13-CC8-C-X1C-X23-C-X6-C	8092.51	8.44
*GhLtpII2*	GhDt09g2478	scaffold4367D09:5900,6181	+	282	93	20	73	C-X7-C-X13-CC8-C-X1C-X23-C-X6-C	8843.68	10.15
*GhLtpII3*	GhDt12g2535	D12:58250975,58251268	–	294	97	30	67	C-X7-C-X13-CC8-C-X1C-X23-C-X6-C	9150.59	8.19
*GhLtpII4*	GhAt12g2427	A12:86743057,86743350	–	294	97	30	67	C-X7-C-X13-CC8-C-X1C-X23-C-X6-C	8729.3	9.42
*GhLtpII5*	GhAt07g1580	A07:60400717,60401007	–	291	96	29	67	C-X7-C-X13-CC8-C-X1C-X23-C-X6-C	8050.37	8.57
*GhLtpII6*	GhDt07g1765	D07:41782012,41782302	–	291	96	29	67	C-X7-C-X13-CC8-C-X1C-X23-C-X6-C	8829.44	7.73
*GhLtpII7*	GhAt12g0503	A12:11958391,11958681	–	291	96	29	67	C-X7-C-X13-CC8-C-X1C-X23-C-X6-C	8353.67	8.18
*GhLtpII8*	GhDt12g0513	D12:9205678,9205968	–	291	96	29	67	C-X7-C-X13-CC8-C-X1C-X23-C-X6-C	9025.4	8.47
*GhLtpII9*	GhDt02g2439	scaffold3867D02:92778,93077	+	300	99	22	77	C-X7-C-X14-CC8-C-X1C-X23-C-X7-C	8809.62	10.09
*GhLtpII10*	GhAt12g0504	A12:12005332,12005622	–	291	96	29	67	C-X7-C-X13-CC8-C-X1C-X23-C-X6-C	8429.97	8.5
*GhLtpII11*	GhDt12g0517	D12:9305273,9305563	–	291	96	29	67	C-X7-C-X13-CC8-C-X1C-X23-C-X6-C	9051.44	8.48
*GhLtpII12*	GhDt12g2180	D12:55001266,55001592	–	327	108	24	84	C-X7-C-X15-CC13-C-X1C-X25-C-X1-C	9111.5	8.87
*GhLtpII13*	GhDt10g1961	D10:54819904,54820308	+	405	134	26	108	C-X7-C-X18-CC13-C-X1C-X24-C-X9-C	9015.7	8.44
*GhLtpII14*	GhAt10g1693	A10:90127094,90127498	+	405	134	26	108	C-X7-C-X18-CC13-C-X1C-X24-C-X9-C	8326.64	8.18
*GhLtpII15*	GhAt10g1692	A10:89958928,89959332	+	405	134	26	108	C-X7-C-X18-CC13-C-X1C-X24-C-X9-C	8254.71	7.72
**Type III**										
*GhLtpIII1*	GhDt12g2484	D12:57779161,57779460	+	300	99	29	70	C-X9-C-X16-CC9-C-X1C-X12-C-X6-C	9262.14	8.9
*GhLtpIII2*	GhAt12g2348	A12:86036418,86036717	+	300	99	29	70	C-X9-C-X16-CC9-C-X1C-X12-C-X6-C	9154.8	9.09
*GhLtpIII3*	GhDt09g2486	scaffold4382D09:53477,53779	+	303	100	34	66	C-X9-C-X16-CC9-C-X1C-X12-C-X6-C	9166.59	8.19
*GhLtpIII4*	GhDt11g0091	D11:859869,860168	+	300	99	36	63	C-X9-C-X16-CC9-C-X1C-X12-C-X6-C	9199.68	8.18
*GhLtpIII5*	GhAt11g0087	A11:836362,836661	+	300	99	36	63	C-X9-C-X16-CC9-C-X1C-X12-C-X6-C	9150.99	9.78
**Type IV**										
*GhLtpIV1*	GhDt01g0687	D01:9691708,9692094	+	387	128	26	102	C-X9-C-X15-CC9-C-X1C-X22-C-X1-C	9465.22	8.89
*GhLtpIV2*	GhAt01g0667	A01:12059506,12059892	+	387	128	26	102	C-X9-C-X15-CC9-C-X1C-X22-C-X1-C	9286	5.18
*GhLtpIV3*	GhDt05g3635	D05:60442682,60443017	+	336	111	29	82	C-X9-C-X15-CC9-C-X1C-X22-C-X9-C	9483.85	8.17
*GhLtpIV4*	GhAt04g0090	A04:1265778,1266128	–	351	116	29	87	C-X9-C-X15-CC9-C-X1C-X22-C-X9-C	9297.25	10.72
*GhLtpIV5*	GhAt13g0411	A13:5661242,5661562	+	321	106	29	77	C-X9-C-X17-CC9-C-X1C-X24-C-X7-C	9271.07	8.14
*GhLtpIV6*	GhDt13g0460	D13:5374248,5374568	+	321	106	29	77	C-X9-C-X17-CC9-C-X1C-X24-C-X7-C	9279.52	5.67
*GhLtpIV7*	GhDt10g1248	D10:22693879,22694184	–	306	101	26	75	C-X9-C-X15-CC9-C-X1C-X24-C-X7-C	9547.01	5.66
*GhLtpIV8*	GhAt10g1243	A10:64635505,64635810	+	306	101	26	75	C-X9-C-X15-CC9-C-X1C-X24-C-X7-C	9431.06	5.48
*GhLtpIV9*	GhAt10g1242	A10:64609157,64609453	+	297	98	23	75	C-X9-C-X15-CC9-C-X1C-X24-C-X7-C	9385.86	8.88
*GhLtpIV10*	GhDt07g1618	D07:31580467,31580796	+	330	109	23	86	C-X9-C-X15-CC9-C-X1C-X24-C-X7-C	9512.95	8.9
*GhLtpIV11*	GhAt07g2213	scaffold1832A07:780824,781120	–	297	98	23	75	C-X9-C-X15-CC9-C-X1C-X24-C-X7-C	9303.12	8.43
*GhLtpIV12*	GhAt11g1319	A11:16995459,16995758	+	300	99	24	75	C-X9-C-X15-CC9-C-X1C-X24-C-X7-C	9437.92	9.06
*GhLtpIV13*	GhDt11g1468	D11:14664570,14664875	+	306	101	26	75	C-X9-C-X15-CC9-C-X1C-X24-C-X7-C	9558.17	8.71
*GhLtpIV14*	GhAt13g0584	A13:13763551,13763940	–	390	129	23	106	C-X9-C-X16-CC12-C-X1C-X24-C-X9-C	9442.98	9.26
**Type V**										
*GhLtpV1*	GhAt12g1415	A12:72762917,72763273	+	357	118	30	88	C-X14-C-X14-CC12-C-X1C-X24-C-X10-C	9831.73	7.44
*GhLtpV2*	GhDt12g1535	D12:46122258,46122614	+	357	118	30	88	C-X14-C-X14-CC12-C-X1C-X24-C-X10-C	10397.2	9.5
*GhLtpV3*	GhDt01g1342	D01:39280406,39280804	–	399	132	30	102	C-X14-C-X14-CC12-C-X1C-X24-C-X10-C	9886.41	6.86
*GhLtpV4*	GhAt01g1193	A01:61929558,61931163	+	366	121	30	91	C-X14-C-X14-CC12-C-X1C-X24-C-X10-C	9559.76	5.13
*GhLtpV5*	GhAt10g2033	A10:98304212,98304625	–	414	137	28	109	C-X14-C-X14-CC11-C-X1C-X24-C-X10-C	9644.15	5.15
*GhLtpV6*	GhDt10g2502	scaffold4400D10:130880,131278	–	399	132	28	104	C-X14-C-X14-CC11-C-X1C-X24-C-X10-C	10389.26	8.15
*GhLtpV7*	GhAt10g2049	A10:98579592,98580011	+	420	139	28	111	C-X14-C-X14-CC11-C-X1C-X24-C-X10-C	9662.65	9.73
*GhLtpV8*	GhDt10g2481	scaffold4398D10:123476,123895	–	420	139	28	111	C-X14-C-X14-CC11-C-X1C-X24-C-X10-C	10264.32	8.09
*GhLtpV9*	GhAt10g2048	A10:98558589,98559002	+	414	137	28	109	C-X14-C-X14-CC11-C-X1C-X24-C-X10-C	9659.07	8.18
*GhLtpV10*	GhDt10g2327	D10:61636841,61637248	–	408	135	28	107	C-X14-C-X14-CC11-C-X1C-X24-C-X10-C	9923.94	8.09
*GhLtpV11*	GhDt04g0626	D04:11317955,11318412	–	348	115	25	90	C-X14-C-X14-CC11-C-X1C-X24-C-X10-C	9903.81	9.32
*GhLtpV12*	GhAt05g3021	A05:77294875,77295338	+	348	115	27	88	C-X14-C-X14-CC11-C-X1C-X24-C-X10-C	9600.08	8.7
*GhLtpV13*	GhDt11g3313	D11:65974290,65974640	–	351	116	28	88	C-X14-C-X14-CC11-C-X1C-X24-C-X10-C	10389.26	8.15
*GhLtpV14*	GhAt11g2927	A11:93115488,93115838	–	351	116	28	88	C-X14-C-X14-CC11-C-X1C-X24-C-X10-C	9696.13	9.18
*GhLtpV15*	GhDt09g1517	D09:42674185,42674529	–	345	114	26	88	C-X14-C-X14-CC11-C-X1C-X24-C-X10-C	9918.57	5.43
*GhLtpV16*	GhAt09g1506	A09:68227589,68227933	–	345	114	26	88	C-X14-C-X14-CC11-C-X1C-X24-C-X10-C	9602.51	9.47
**Type VI**										
*GhLtpVI1*	GhAt12g1935	A12:82111877,82112218	+	342	113	23	90	C-X10-C-X12-CC9-C-X1C-X22-C-X9-C	10461.43	10.17
*GhLtpVI2*	GhDt12g2116	D12:54193081,54193422	+	342	113	26	87	C-X10-C-X12-CC9-C-X1C-X22-C-X9-C	10502.07	4.8
*GhLtpVI3*	GhDt13g1254	D13:38598772,38600424	+	324	107	27	80	C-X10-C-X12-CC9-C-X1C-X22-C-X9-C	10533.51	8.43
*GhLtpVI4*	GhDt07g1207	D07:18392595,18393016	–	330	109	21	88	C-X10-C-X16-CC9-C-X1C-X22-C-X9-C	10470.61	8.8
**Type VIII**										
*GhLtpVIII1*	GhDt08g1864	D08:56168875,56169490	+	417	138	23	115	C-X6-C-X14-CC12-C-X1C-X25-C-X8-C	10715.97	8.58
*GhLtpVIII2*	Gh_Sca022247g01	scaffold22247:295,910	–	417	138	23	115	C-X6-C-X14-CC12-C-X1C-X25-C-X8-C	10576.07	5.43
*GhLtpVIII3*	GhAt08g1556	A08:93518570,93519184	+	417	138	23	115	C-X6-C-X14-CC12-C-X1C-X25-C-X8-C	10637.58	8.81
*GhLtpVIII4*	GhDt03g1537	D03:44580189,44581423	–	426	141	25	116	C-X6-C-X14-CC12-C-X1C-X25-C-X8-C	10675.55	8.64
*GhLtpVIII5*	GhAt03g1989	scaffold500A03:40091,41395	–	426	141	25	116	C-X6-C-X14-CC12-C-X1C-X25-C-X8-C	10631.15	5.16
*GhLtpVIII6*	GhDt12g2189	D12:55096494,55096981	+	360	119	18	101	C-X6-C-X14-CC12-C-X1C-X25-C-X8-C	10746	8.58
*GhLtpVIII7*	GhAt12g2013	A12:83061591,83061929	+	339	112	23	89	C-X6-C-X14-CC12-C-X1C-X25-C-X8-C	10640.2	4.28
*GhLtpVIII8*	GhDt03g1533	D03:44563981,44565739	–	384	127	25	102	C-X6-C-X14-CC12-C-X1C-X27-C-X8-C	10664.5	8.79
*GhLtpVIII9*	GhAt03g1987	scaffold500A03:25416,26159	–	339	112	25	87	C-X6-C-X14-CC12-C-X1C-X25-C-X8-C	10583.15	6.02
*GhLtpVIII10*	GhDt03g1535	D03:44570607,44570975	+	369	122	20	102	C-X6-C-X14-CC12-C-X1C-X25-C-X8-C	10669.13	4.07
*GhLtpVIII11*	GhAt03g1988	scaffold500A03:33691,34059	+	369	122	20	102	C-X6-C-X14-CC12-C-X1C-X25-C-X8-C	10597.79	8.8
**Type IX**										
*GhLtpIX1*	GhDt08g1612	D08:50811277,50811732	–	354	117	38	79	C-X13-C-X15-CC9-C-X1C-X22-C-X6-C	10820.71	8.64
*GhLtpIX2*	GhAt08g1322	A08:85793075,85793524	–	450	149	38	111	C-X13-C-X15-CC9-C-X1C-X22-C-X6-C	10747.88	8.59
*GhLtpIX3*	GhAt12g2579	scaffold3294A12:7344,7679	+	336	111	28	83	C-X13-C-X15-CC9-C-X1C-X22-C-X6-C	10747.88	8.59
**Type XI**										
*GhLtpXI1*	GhAt13g0036	A13:372098,372436	+	339	112	26	86	C-X9-C-X17-CC13-C-X1C-X25-C-X7-C	11320.35	9.04
*GhLtpXI2*	GhDt13g0051	D13:431484,431819	+	336	111	25	86	C-X9-C-X17-CC13-C-X1C-X25-C-X7-C	12087.7	4.16
*GhLtpXI3*	GhAt13g0035	A13:367676,368014	–	339	112	26	86	C-X9-C-X17-CC13-C-X1C-X25-C-X8-C	11306.2	5.58
*GhLtpXI4*	GhAt07g0235	A07:2836735,2837103	–	369	122	25	97	C-X9-C-X18-CC13-C-X1C-X24-C-X9-C	10975.1	9.13
*GhLtpXI5*	GhDt07g0293	D07:2996520,2996900	–	381	126	25	101	C-X9-C-X18-CC13-C-X1C-X24-C-X9-C	11327.48	9.1
*GhLtpXI6*	GhDt13g1963	D13:55015573,55015971	+	399	132	26	106	C-X9-C-X18-CC13-C-X1C-X24-C-X9-C	12096.75	4.52
*GhLtpXI7*	GhAt13g1604	A13:74895665,74896063	+	399	132	26	106	C-X9-C-X18-CC13-C-X1C-X24-C-X9-C	11327.48	9.1
*GhLtpXI8*	GhDt12g1005	D12:35698319,35698696	–	378	125	22	103	C-X9-C-X18-CC13-C-X1C-X24-C-X9-C	12022.63	4.52
*GhLtpXI9*	GhAt12g0916	A12:59270460,59270840	–	381	126	22	104	C-X9-C-X18-CC13-C-X1C-X25-C-X9-C	11264.4	9.06
*GhLtpXI10*	GhDt08g1843	D08:55412918,55413304	–	387	128	26	102	C-X9-C-X20-CC13-C-X1C-X24-C-X9-C	11332.31	8.88
*GhLtpXI11*	GhAt08g1542	A08:92708059,92708445	–	387	128	26	102	C-X9-C-X20-CC13-C-X1C-X24-C-X9-C	10996.86	4.04
*GhLtpXI12*	GhAt11g0232	A11:2146622,2147029	+	408	135	26	109	C-X9-C-X17-CC13-C-X1C-X24-C-X9-C	11140.01	4.27
*GhLtpXI13*	GhDt11g0246	D11:2037237,2037644	+	408	135	26	109	C-X9-C-X17-CC13-C-X1C-X24-C-X9-C	11422.24	4.69
*GhLtpXI14*	GhDt11g0253	D11:2098320,2098709	–	390	129	26	103	C-X9-C-X18-CC13-C-X1C-X24-C-X9-C	11651.91	9.34
*GhLtpXI15*	GhDt11g0251	D11:2080167,2080577	–	411	136	26	110	C-X9-C-X18-CC13-C-X1C-X24-C-X9-C	11599.68	8.84
*GhLtpXI16*	GhAt11g0236	A11:2216177,2216587	–	411	136	26	110	C-X9-C-X18-CC13-C-X1C-X24-C-X9-C	11198.43	8.8
*GhLtpXI17*	GhDt11g0254	D11:2108602,2109012	–	411	136	26	110	C-X9-C-X18-CC13-C-X1C-X24-C-X9-C	11753.95	9.16
*GhLtpXI18*	GhAt11g0239	A11:2241886,2242296	–	411	136	26	110	C-X9-C-X18-CC13-C-X1C-X24-C-X9-C	11249.31	8.8
*GhLtpXI19*	GhDt11g0252	D11:2085276,2085674	–	399	132	26	106	C-X9-C-X18-CC13-C-X1C-X24-C-X9-C	11616.47	8.83
*GhLtpXI20*	GhAt11g0237	A11:2221458,2221856	–	399	132	26	106	C-X9-C-X18-CC13-C-X1C-X24-C-X9-C	11240.97	5.48
*GhLtpXI21*	GhDt11g0250	D11:2074032,2074424	–	393	130	26	104	C-X9-C-X18-CC13-C-X1C-X24-C-X9-C	11598.74	9.06
*GhLtpXI22*	Gh_Sca005825g01	scaffold5825:3233,3625	–	393	130	26	104	C-X9-C-X18-CC13-C-X1C-X24-C-X9-C	10820.71	8.64
*GhLtpXI23*	GhDt11g0249	D11:2069748,2070152	–	405	134	26	108	C-X9-C-X18-CC13-C-X1C-X24-C-X9-C	11483.3	8.95
*GhLtpXI24*	GhDt11g0248	D11:2047537,2047941	–	405	134	26	108	C-X9-C-X18-CC13-C-X1C-X24-C-X9-C	11458.29	10.1
*GhLtpXI25*	GhAt11g0234	A11:2156230,2156634	–	405	134	26	108	C-X9-C-X18-CC13-C-X1C-X24-C-X9-C	11194.26	8.64
*GhLtpXI26*	GhAt11g0233	A11:2153158,2153565	–	408	135	26	109	C-X9-C-X18-CC13-C-X1C-X24-C-X9-C	11142.3	8.86
*GhLtpXI27*	GhDt11g0247	D11:2044676,2045089	–	414	137	26	111	C-X9-C-X18-CC13-C-X1C-X24-C-X9-C	11447.61	8.81
*GhLtpXI28*	GhDt08g1844	D08:55438196,55438594	+	399	132	26	106	C-X9-C-X18-CC13-C-X1C-X24-C-X9-C	11346.34	8.88
*GhLtpXI29*	GhAt08g1543	A08:92737721,92738119	+	399	132	26	106	C-X9-C-X18-CC13-C-X1C-X24-C-X9-C	11108.3	8.81
*GhLtpXI30*	GhDt08g1846	D08:55481342,55481734	+	393	130	26	104	C-X9-C-X18-CC13-C-X1C-X24-C-X9-C	11386.19	4.69
*GhLtpXI31*	GhDt08g1845	D08:55457004,55457402	+	399	132	26	106	C-X9-C-X18-CC13-C-X1C-X24-C-X9-C	11346.34	8.88
*GhLtpXI32*	GhAt12g2002	A12:82919563,82919982	+	420	139	26	113	C-X9-C-X19-CC13-C-X1C-X24-C-X9-C	11269.07	10.27
*GhLtpXI33*	GhDt12g2178	D12:54989587,54990000	+	414	137	26	111	C-X9-C-X19-CC13-C-X1C-X24-C-X9-C	12076.3	8.44
*GhLtpXI34*	Gh_Sca071160g01	scaffold71160:10,423	–	414	137	26	111	C-X9-C-X19-CC13-C-X1C-X24-C-X9-C	10863.37	6.31
**nsLTPy**										
*nsLTPy1*	GhAt09g1626	A09:69945165,69945455	+	291	96	25	71	C-X8-C-X12-CC8-C-X1C-X23-C-X7-C	12152.77	4.11
*nsLTPy2*	GhAt09g1629	A09:69958407,69958697	+	291	96	25	71	C-X8-C-X12-CC8-C-X1C-X23-C-X7-C	12262.29	8.96
*nsLTPy3*	GhDt09g1721	D09:44973379,44973669	+	291	96	25	71	C-X8-C-X12-CC8-C-X1C-X23-C-X7-C	12428.51	8.96
*nsLTPy4*	GhDt09g1723	D09:44986600,44986890	+	291	96	25	71	C-X8-C-X12-CC8-C-X1C-X23-C-X7-C	12525.63	9.07
*nsLTPy5*	GhDt09g1722	D09:44983127,44983417	+	291	96	25	71	C-X8-C-X12-CC8-C-X1C-X23-C-X7-C	12492.48	9.47
*nsLTPy6*	GhAt09g1625	A09:69919600,69919890	+	291	96	25	71	C-X8-C-X12-CC8-C-X1C-X23-C-X7-C	12096.75	4.52
*nsLTPy7*	GhAt09g1628	A09:69954924,69955217	+	294	97	25	72	C-X8-C-X12-CC8-C-X1C-X23-C-X7-C	12253.28	8.95
*nsLTPy8*	GhAt09g1627	A09:69948720,69949010	+	291	96	25	71	C-X8-C-X12-CC8-C-X1C-X23-C-X7-C	12178.2	8.31

a AA, number of amino acids;

b SP, signal peptide;

c MP, mature protein;

d ECM, eight cysteine motif;

e MW, molecular weight in Dalton;

f *pI, isoelectric point*.

**Figure 1:**
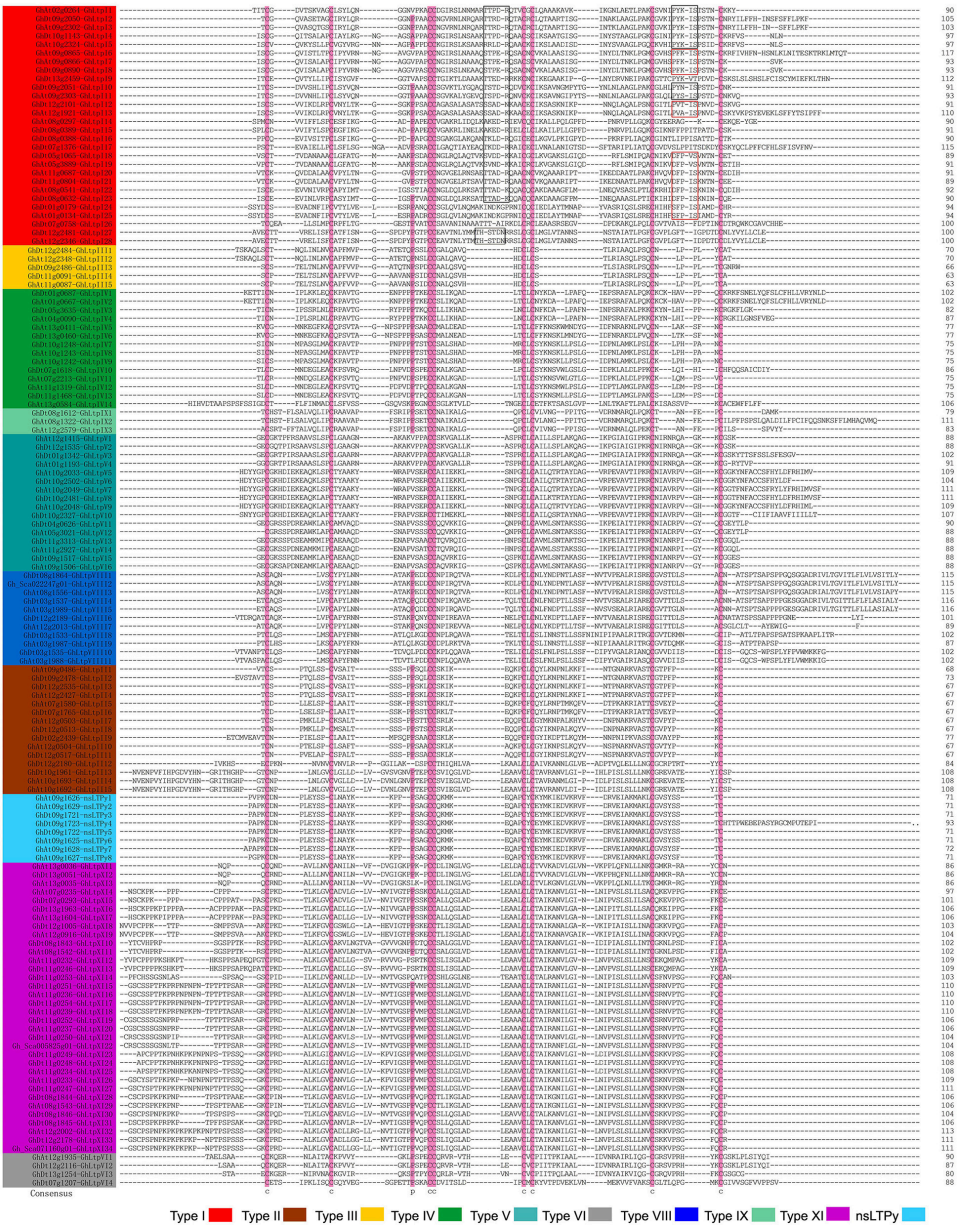
Multiple sequence alignment of GhLtp proteins. The gene ID and names of different types of GhLtps are presented with different colors. The conserved cysteine residues are marked against pink backgrounds. Consensus residues are marked by rectangles.

The sequence similarity method (Boutrot et al., 2008) was employed to classify the identified GhLtps and our results showed that the 138 GhLtps could be divided into 10 groups after multiple sequence alignments (Table 1 and Supplemental Table S2). The results showed that the ECMs were highly conserved in all of the 138 GhLtps, which could form four disulfide bonds to stabilize the tertiary structure of hydrophobic cavity (Figure 1). Different from rice, wheat, rape, and *Arabidopsis*, in which the majority of nsLTPs belongs to type I or II (Boutrot et al., 2008; Li et al., 2014), type XI is the largest group in *G. hirsutum* (Figure 1 and Table 1). The flanking amino acid residues of each CXC motif were conserved within the members of the same group, except for type I. A more conserved consensus pentapeptide Thr/Ser-X1-X2-Asp-Arg/Lys and a more variable one Pro-Tyr-X-Ile-Ser were found in type I GhLtps as reported previously (Douliez et al., 2000; Li et al., 2014). As small molecules, nsLTPs lack supernumerary sequences at C/N-terminal or contain a short C-terminal tail. However, GhLtpXIs contained a relatively long N-terminal cap.

The molecular weight (MW) and theoretical pI (isoelectric point) of each GhLtp were calculated and summarized in Table 1. Low MW is a common characteristic of plant nsLTPs and very few nsLTPs were found with a MW above 11 kDa in rice, rape and *Arabidopsis*. Interestingly, all GhLtpXIs displayed high MW (about 11–13 kDa). The high MWs of all the type XI members were due to the presence of supernumerary amino acid residues located at the N-terminal.

### Phylogenetic and sequence analysis of nsLTPs

**Figure 2:**
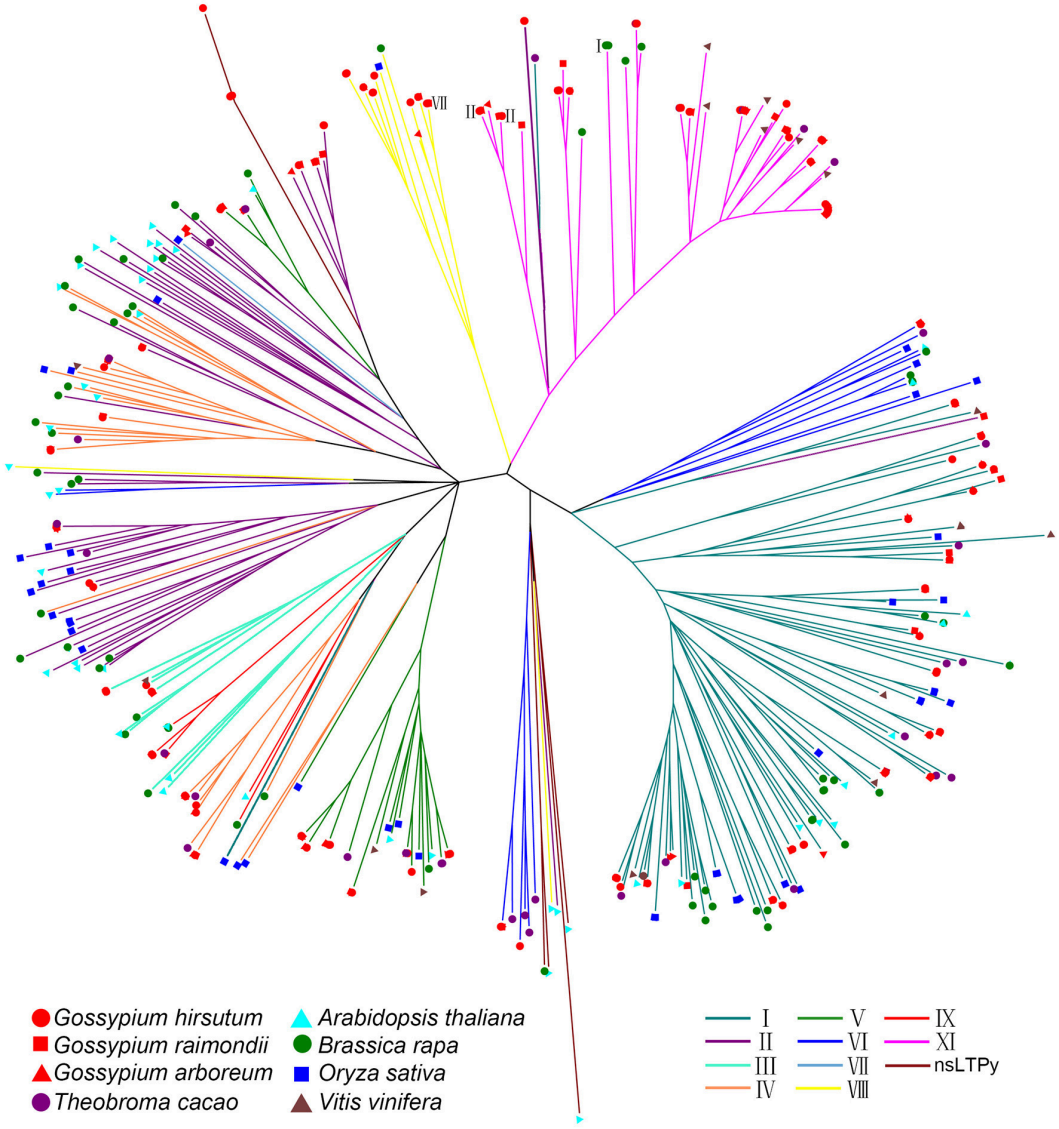
Phylogenetic tree of nsLtps in eight species. The full length of mature protein sequences of nsLTPs from *G. hirsutum, G. arboreum, G. raimondii, Arabidopsis, B. rapa, Th. cacao, O. sativa*, and *V. vinifera* were used to construct the phylogenetic tree using a Neighbor-Joining method. Lines of different colors represent classification of nsLTPs. Different species were marked at the end of the lines. Greek numerals present the corresponding type of genes that cannot be displayed by lines.

Type XI nsLPTs are rare in plants. None of them was identified in rice and wheat, and only two and three nsLTPs belong to this group in *Arabidopsis* and *B. rapa*, respectively (Boutrot et al., 2008; Li et al., 2014). On the contrary, type XI contained most members in *G. hirsutum*. To study the evolution of cotton *nsLTP* genes, this gene family was additionally identified and classified from *G. arboreum, G. raimondii, Th. cacao* and *V. vinifera* using the same method described above (Supplemental Table S3). An unrooted phylogenetic tree was subsequently built with nsLTP proteins from 8 species including *G. hirsutum, G. arboreum, G. raimondii, A. thaliana, B. rapa, Th. cacao, O. sativa*, and *V. vinifera* with neighbor-joining method (Figure 2). None of these proteins formed distinct monophyletic clusters. Type I was the largest group in plants and most sequences of this group formed a separated cluster in the tree. Cotton species have evolved a large number of type XI genes though this group is also identified in grape, cacao and rape. Two type I genes (Bra024938 and Tc11g016320) and five cotton type II genes (Ga3164g39880 and GhLtpII12-15) were close to type XI genes, indicating that cotton type XI genes maybe evolved from type I or II genes. Most importantly, only type XI sequences formed a specific cluster in the tree, indicating that these genes shared a common ancestor.

To further study the phylogenetic organization of the GhLtpXIs, a phylogenetic tree was constructed using Maximum-likehood inference from the alignment of respective 138 and 53 protein sequences of *G. hirsutum* and *Arabidopsis*. The results showed that all the nsLTPs can be divided into four clusters from A-D (Supplemental Figure S1). All the type I and III GhLtps belonged to cluster C. Type V, VIII, and IX GhLtps fell into cluster D, and cluster A contained all members of type VI and XI. Phylogenetic analysis had shown that the same type AtLtps constituted monophyletic groups except for the type II (Boutrot et al., 2008; Li et al., 2014). Consistently, GhLtpII12-15 were more distantly related to other type II GhLtps. GhLtpII13, GhLtpII14, and GhLtpII15 were more close to GhLtpXIs, while GhLtpII12 was more related to GhLtpVIs and GhLtpXI1/2/3.

Gene structures of the GhLtps were obtained by comparing the predicted CDS with their corresponding genomic DNA sequences (Supplemental Figure S2). The results showed that only 16 *GhLtps* had introns. Among these *GhLtps*, six *GhLtps* were interrupted by two introns, and the others were interrupted by a single intron. According to the previous studies in some other plants, 35 out of 52 *OsLtps*, 25 out of 51 *AtLtps*, and 17 out of 63 *BrnsLtps* contain introns (Boutrot et al., 2008; Li et al., 2014). These results revealed that the percentage of *GhLtps* lacking introns was much higher among rice, *Arabidopsis, B. rapa*, and *G. hirsutum*, and the diverse distribution of intronic regions was quite low.

### Chromosomal localization and gene duplication of *GhLtps*

**Figure 3:**
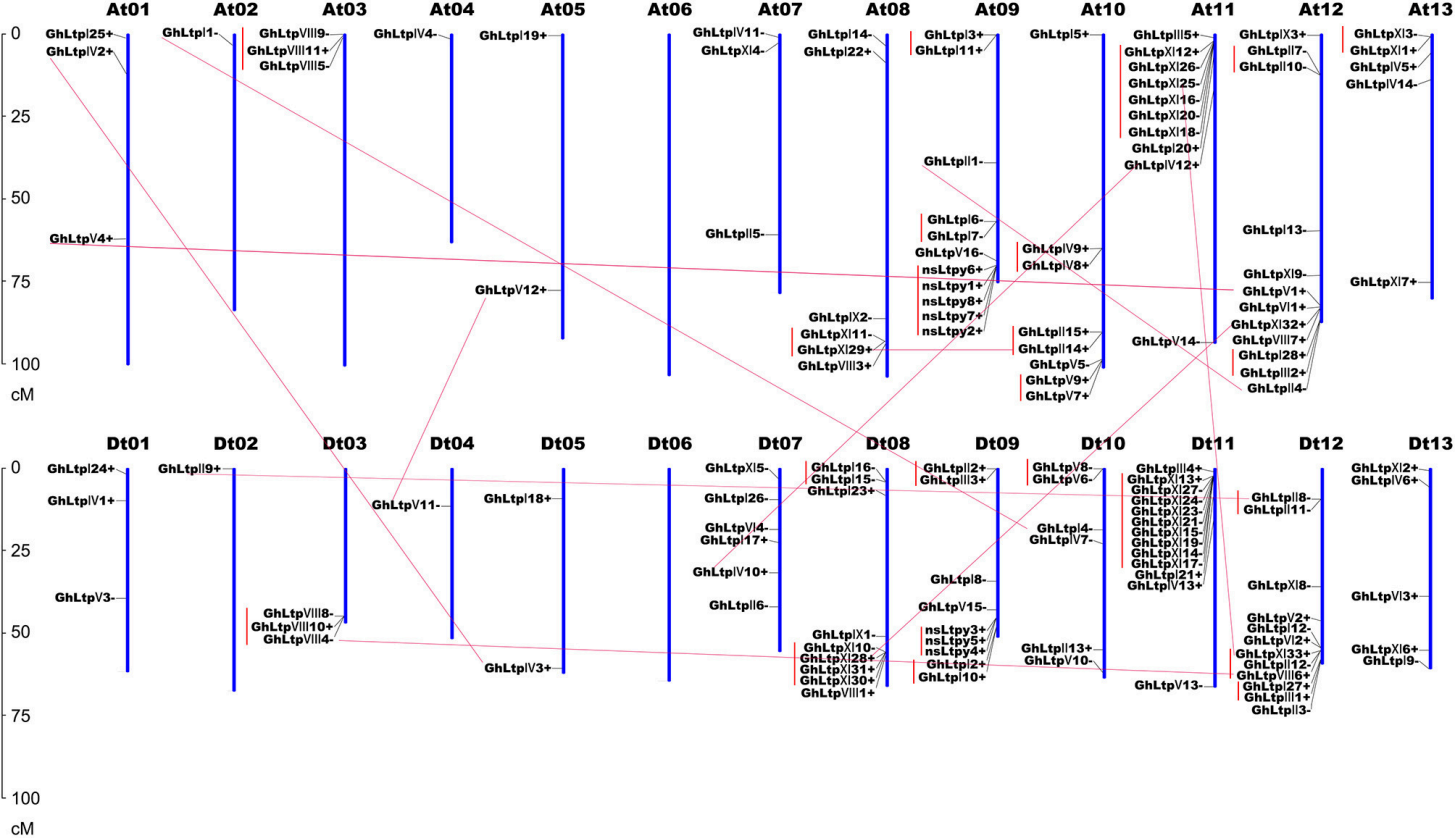
Distribution of *GhLtps* in 26 chromosomes. Chromosome numbers are indicated above each vertical bar. The scale represents centimorgan (cM). The segmental duplicated gene pairs are connected with red lines. The tandem duplicated gene clusters are marked in red perpendicular lines.

To determine the chromosomal distribution of *GhLtps*, the approximate position of each *GhLtp* was marked on the physical map of the 26 *G. hirsutum* chromosomes (Figure 3). It was found that the majority of the *GhLtp* genes located at the ends of the chromosomes. Chromosome Dt11 harbored the most *GhLtps* with 13 genes including 9 *GhLtpXIs* and none of the *GhLtps* located on chromosome At06 and Dt06. Since *G. hirsutum* is tetraploid with subgenome At and Dt, comparison was performed between each pair of homologous chromosomes. Ignoring three genes that are unable to locate, the *GhLtps* almost equally positioned on each genome and the number of genes on chromosomes At and its relative homologous chromosome Dt was uniform or close. Additionally, the distribution patterns of *GhLtps* were quite similar within each pair of homologous chromosomes, which would probably result from complex evolutional process including recombination, DNA exchanges and gene duplications after merge of genome A and D. These results showed that *GhLtps* distributed congruently within subgenome At and Dt.

Gene duplication events including segmental and tandem duplications were thought to be essential for the expansion of gene family in the genome (Maere et al., 2005) and thus gene duplication events of *nsLTP* family were investigated in *G. hirsutum*. A total of 32 duplication events were found, including 9 segmental duplication pairs and 23 tandem duplication pairs (Figure 3 and Supplemental Table S4). The duplication events were mainly concentrated in the same subfamily except for three pairs of tandem duplication (*GhLtpII2*/*III3, GhLtpI28*/*III2*, and *GhLtpXI33*/*II12*/*VIII6*). It was worthy to note that tandem duplication pairs distributed in the proximate locations of each paralogous blocks and the expansion of type XI group was largely contributed by tandem duplication. We subsequently calculated the non-synonymous to synonymous substitution ratio (Ka/Ks) for each duplicated *GhLtp* gene pairs. Several duplication pairs of *GhLtpXIs* displayed larger Ks values and these genes constituted four gene clusters located in chromosomes 08 and 11 in subgenome At and Dt, implying an early divergence time of these *GhLtpXIs*. Additionally, most Ka/Ks ratios were below 1, except for five duplication pairs (Supplemental Table S4), suggesting that *GhLtps* had mainly experienced strong purifying selection pressure with limited functional divergence. These results revealed that *GhLtpXIs* expanded early on chromosome At11 and Dt11 and the functions of the duplicated *GhLtps* did not diverge much during evolution.

### Transcriptional analysis of *GhLtps*

In this study, expression of *GhLtps* were analyzed in various organs/tissues (Supplemental Figure S3). It was worthy to point out that all the type nsLTPy genes showed specific expression in stamen, while other group members did not show any expression preference. Thirty four genes showed abundant transcription profile during fiber development and nearly 30% of them pertained to type XI.

Due to the irreplaceable economical value of cotton fibers, breeders have been making persistent efforts to develop various types of cotton (*Gossypium spp*.) with desirable fiber characteristics, which provides materials for investigation of the regulation of fiber development (Han et al., 2016). To further elucidate the function of *GhLtps* during fiber development, the expression of *GhLtps* in cultivars HY405, CCRI8, and ND601 was analyzed using our RNA-seq data. It was noted that 110 genes out of 138 *GhLtps* were expressed during fiber development (Supplemental Figure S4). The other 28 *GhLtps* with scarcely any transcripts covered each group. *GhLtpI1*/*10*/*11* and *GhLtpXI32* were highly transcribed during fiber development. And seven *GhLtpXIs* out of 18 *GhLtps* demonstrated high transcripts only in fiber initiation and early elongation stage. Further, the 138 *GhLtps* could be clustered into four groups based on the expression trend during fiber development (Supplemental Figure S5). The majority of *GhLtps* were expressed in the fiber initiation stage, and kept a relatively high expression level during the early stage of elongation. It was noticeable that the expression of nearly half of the *GhLtps* decreased gradually, suggesting important roles in cotton fiber initiation and elongation which are essential for lint fiber formation. Additionally, over 60% of *GhLtpXIs* displayed higher expression during fiber initiation and early elongation. It seemed that type XI *GhLtps* were important for fiber initiation and elongation.

**Figure 4:**
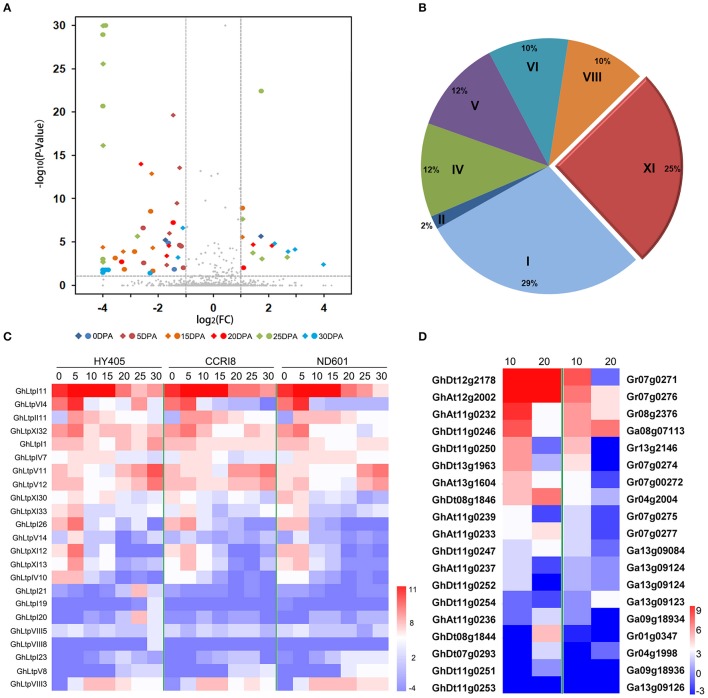
Differential expression analysis of *GhLtps*. **(A)** Volcano plot shows expression comparison between HY405 vs. CCRI8 and HY405 vs. ND601 represented by round and square points, respectively. FC stands for fold change. **(B)** Pie chart shows the proportion of gene types in differentially expressed *GhLtps*. **(C)** Heatmap shows expression of differentially expressed genes between cultivars with longer and shorter fibers. Data shown were log_2_-transformed RPKM. Developmental stages of fiber are indicated in days post anthesis (DPA) above. The color bar represents the relative expression level. **(D)** Heatmap shows expression of differentially expressed *GhLtps* compared with their orthologs in *G. raimondii* or *G. arboreum*. Data shown were log_2_-transformed FPKM of each gene which was quantified using RNA-seq data downloaded from CottonFGD. Developmental stages of fiber are indicated in days post anthesis (DPA) above. The color bar represents the relative expression level.

Further comparison of the transcription level was made between longer (HY405) and shorter (CCRI8 and ND601) fibers. The results between comparison of HY405 vs. CCRI8 and HY405 vs. ND601 were consistent (Figure 4A). More *GhLtps* were significantly activated in cultivar HY405 with longer fibers, especially at 5, 15, and 25 DPA (Figure 4A). Among these genes, *GhLtpIs* and *GhLtpXIs* occupied 54% proportion (Figure 4B). The transcripts of *GhLtpI26, V14, VI4, XI12, XI13, XI30, XI32*, and *XI33* dramatically accumulated in long-fibered cultivars when fiber started to elongate (Figures 4A,C), suggesting a possible contribution of *GhLtps* (especially *GhLtpXIs*) to fiber length improvement.

After its divergence from an ancestor shared with *Th. cacao* (Carvalho et al., 2011), the cotton lineage evolved into D-genome and A-genome. However, spinnable fiber only evolved in the A-genome and was further elongated after the merger of A and D genome (Paterson et al., 2012). In order to further clarify the function of *GhLtpXIs* in fiber development, the expression of cotton type XI genes was analyzed. Firstly, orthologs of *GhLtpXIs* were identified from D-genome and A-genome according to the sequence similarity, which was further confirmed by the syntenic relationships between diploid and tetraploid cotton species identified previously (Supplemental Figure S6) (Zhang et al., 2015). Ten type XI genes in A and D genome lost during evolution and 4 pairs of non-reciprocal DNA exchanges were identified including Gr13g0054/GhAt13g0035, GhAt11g0237/GhDt11g0252, GhAt11g0234/GhDt12g1005, and GhDt12g1005/GhAt12g0916. Then the expression of orthologous gene pairs in ovules was compared at 10 and 20 DPA, respectively, using the downloaded transcriptome data. The results demonstrated that the transcriptional level of most *GhLtpXIs*, discarding 12 *GhLtpXIs* with scarce transcripts, significantly varied from their diploid progenitors (Figure 4D), among which the expression of 15 *GhLtpXIs* varied at 10 DPA and 12 varied at 20 DPA. It was notable that the expression of most *GhLtpXIs* was higher than their diploid progenitors except for *GhLtpXI14-17*. Especially, expression of *GhLtpXI32* and *GhLtpXI33* was extremely higher in tetraploid cotton than their orthologs in diploid cotton. Additionally, transcripts of *GhLtpXI6* and *GhLtpXI12* were dramatically abundant during fiber elongation in tetraploid cotton and *GhLtpXI27, GhLtpXI28*, and *GhLtpXI30* increased sharply at 20 DPA.

The previous studies showed that polar lipids increased and reached the maximum level and were incorporated into the cell wall from 3 to 20 DPA (Wan et al., 2005; Edstam et al., 2013; Kumar et al., 2013). Our results suggested that *GhLtps*, especially *GhLtpXIs* play important roles during fiber elongation. Those differentially expressed *GhLtps* are likely to delivering lipids to the outer integument of cotton ovules (Edstam et al., 2013) and thus contribute to the variance of fiber length.

### *GhLtpXIs* are involved in fiber development

**Figure 5:**
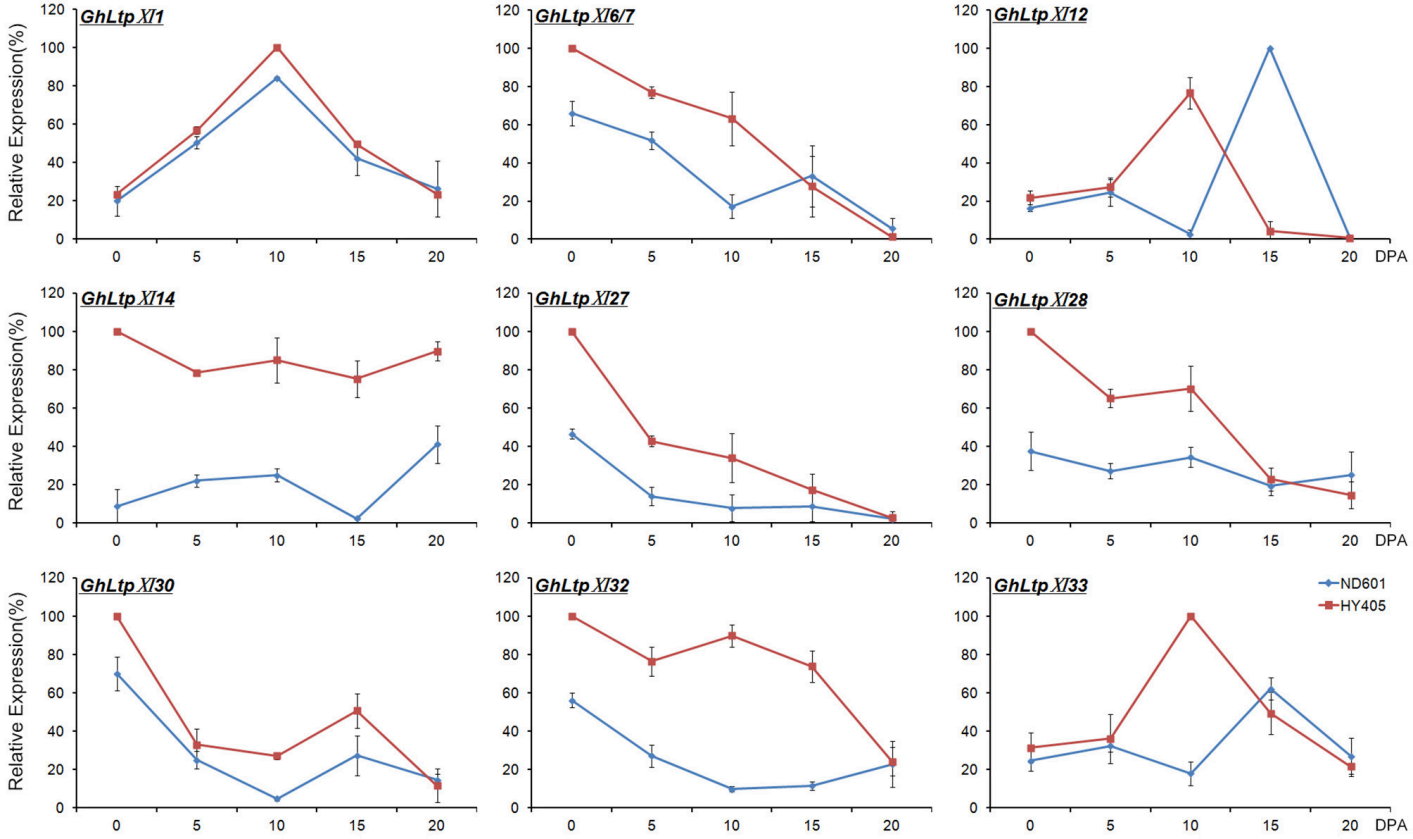
Temporal expression of nine *GhLtpXIs* in developing fibers. Ovules were collected on the day of anthesis and fibers were harvested on 5, 10, 15, and 20 DPA. Gene expression levels determined by qPCR were normalized to *UBQ14* expression and shown as relative values to the maximal gene expression levels set at 100%. Error bars indicate SD of three biological replicates.

Based on the analysis of the RNA-seq data, it is possible that the considerably expanded *GhLtpXIs* play important roles in different fiber developmental stages. On account of this, we selected nine genes to verify their expression by qPCR (Figure 5). The expression trend of the selected genes were consistent with RNA-seq data except for *GhLtpXI12* and *GhLtpXI33*, which would be due to that samples at 5 DPA used for RNA-seq and qRT-PCR were different. All of these *GhLtpXIs* showed higher transcription levels in cultivar HY405 that produces longer fibers. The expression profile of each gene varied in different stages of fiber development, suggesting different roles of *GhLtpXIs*. *GhLtpXI27, GhLtpXI28*, and *GhLtpXI30* exhibited highest transcripts at 0 DPA and decreased dramatically at 5 DPA, revealing a role in fiber initiation. The expression of *GhLtpXI6/7* decreased gradually during fiber elongation. Noticeably, *GhLtpXI6/7* maintained a high level in HY405 until 10 DPA when its transcription in ND601 has decreased to a relatively low level, and *GhLtpXI14* and *GhLtpXI32* demonstrated a constantly high level during fiber elongation in HY405, which would probably contribute to long fiber quality of the cultivar. *GhLtpXI1, GhLtpXI12*, and *GhLtpXI33* showed a transcriptional peak during fiber elongation. The expression trend of *GhLtpXI1* was consistent in both cultivars with a higher expression in HY405. Though the expression of *GhLtpXI12* was comparable in two cultivars, its expression peaked earlier in HY405 at 10 DPA that is essential to fiber elongation. There is also an advanced expression peak of *GhLtpXI33* in HY405 with a significantly abundant transcripts compared with that in ND601. It might be another important effecter on fiber length.

**Figure 6:**
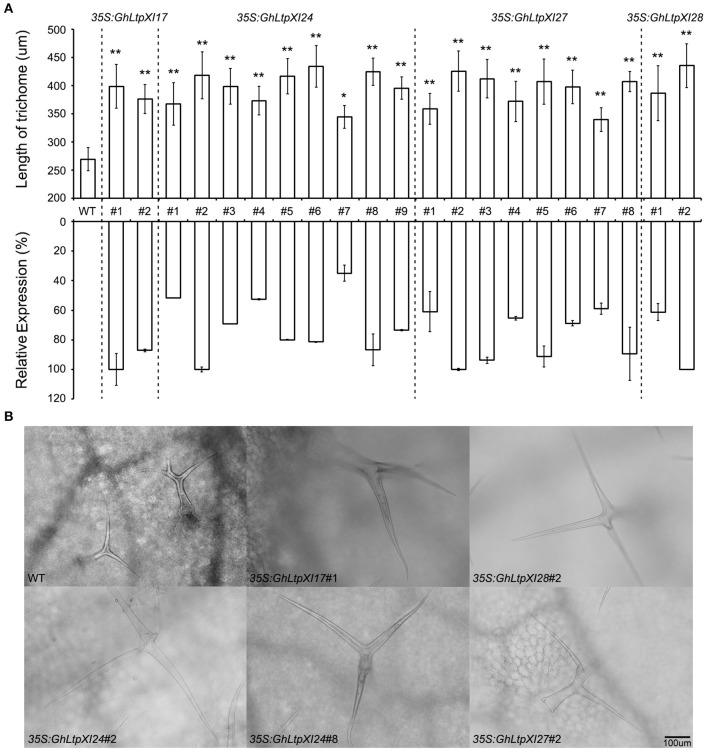
*GhLtpXIs* regulated trichome development in *Arabidopsis*. **(A)** Upregulation of *GhLtpXIs* in independent overexpression transgenic plants correlated to the length of trichomes on the surface of mature rosette leaves. Trichome length was measured under microscope and calculated for average of 30~50 trichomes. Asterisks indicate the significant difference in relation to WT (*T*-test, ^*^p < 0.05, ^**^p < 0.01). Expression of *GhLtpXIs* was determined by qPCR in leaves of each independent transgenic plants. Transcripts of *GhLtpXIs* were undetectable in WT. Results were normalized against the expression of *TUB2*. The maximum expression of each *GhLtpXI* is set as 100%. Error bars indicate SD. **(B)** Overexpressing of *GhLtpXIs* in transgenic plants promoted trichome length. Trichomes were observed under microscope after decolorization of leaves. The bar represents 100 μm.

It is believed that the regulatory mechanism is shared by fiber and leaf trichome development (Kim and Triplett, 2001; Qin and Zhu, 2011; Lei et al., 2014), and *Arabidopsis* continues to serve as a useful experimental system for dissecting the mechanisms of cotton fiber development (Guan et al., 2014; Shangguan et al., 2016; Ma et al., 2018). Since *GhLtpXIs* considerably expanded in *Gossypium* species, several members with early divergent time were cloned and ectopically expressed in *Arabidopsis* to further verify the function of *GhLtpXIs* in fiber development. Expression analysis of T1 transgenic plants revealed that these cotton genes were successfully expressed in *Arabidopsis* (Figure 6A). The trichomes of the mature rosette leaves were observed under microscope. The results showed that the trichome morphology was not affected by overexpressing of different *GhLtpXIs*, whereas the trichome length of all the transgenic plants was significantly longer than that of the WT plants (Figure 6B), and the improvement of trichome length was positively correlated to the expression of *GhLtpXIs*. These results suggested that *GhLtpXIs* function to promote trichome elongation. Cotton fiber which is derived from the epidermal cells is a single-celled trichome of seed and thus these *GhLtpXIs* might probably regulate fiber elongation.

## Discussion

### Type XI nsLTPs expanded considerably in *gossypium* species

Plant nsLTPs are characterized by the ECM backbone forming a stabilized hydrophobic cavity and could be classified into 11 groups including I, II, III, IV, V, VI, VII, VIII, IX, XI, and nsLTPy according to the number of flanking amino acids within the conserved ECM domain (Kader, 1996; Carvalho and Gomes, 2007; Boutrot et al., 2008; Li et al., 2014). The nsLTP family has been identified in *Arabidopsis*, rice, rape, maize, and sorghum in previous studies (Boutrot et al., 2008; Li et al., 2014; Wei and Zhong, 2014) and in *G. hirsutum, G. arboreum, G. raimondii, Th. Cacao*, and *V. vinifera* in this study (Supplemental Table S3). As an ancient species, *V. vinifera* genome contained 3 type XI genes, and this group is likely to disappear evolutionally because of the lack of type XI genes in rice and *Arabidopsis*. Although several type XI genes were identified in cacao, grape and rape, type I or II consistently possess most members in grape, cacao, rape, rice and *Arabidopsis*. It is interesting that cotton type XI nsLTPs harbor an extra N-terminal cap and are larger in molecular weight, which are different from other nsLTPs. These outcomes reveal that type XI nsLTPs specifically expanded in the *Gossypium* species (Figure 2).

Cotton type XI genes were close to type II genes in the phylogenetic tree and duplication pairs were found between *GhLtpXIs* and *GhLtpIIs* (Figures 2, 3 and Supplemental Table S4). The special protein structure of GhLtpXIs was only similar to GhLtpII13-15 (Figure 1). These findings imply that cotton type XI genes might diverge from type II genes. A large amount of tandem duplication events was found within *GhLtpXIs* (Figure 3), suggesting an essential contribution to the tremendous expansion of type XI genes.

### *GhLtpXIs* regulate fiber development

Considered as an ideal model for cell elongation, cotton fiber is the single-celled seed trichome developed from the outer integument of ovule and cotton fiber quality is quantitative traits controlled by multiple genes. Due to their economic importance and biological feature, cotton fiber development has been the subject of much scientific interest. Two decades ago, scientists have cloned nsLTPs from fibers and believed that they should play roles during fiber development (Ma et al., 1995, 1997; Liu et al., 2000; Orford and Timmis, 2000). Later, 12 nsLTPs were found highly expressed during fiber elongation in upland cotton fibers compared with fuzzless mutant ovules (Ji et al., 2003). However, little progress was achieved on how these small proteins affect fiber development. The complement of whole genome sequence of *G. hirsutum* (Li et al., 2015; Zhang et al., 2015) facilitates the study of gene families contributing to fiber development. In the present study, 138, 65, and 70 nsLTPs were strictly identified from *G. hirsutum, G. arboretum*, and *G. raimondii*, respectively. RNA-seq data suggested that most *GhLtps* were transcribed during fiber development and some *GhLtpXIs* displayed much abundant transcripts during fiber initiation and elongation (Figure 5 and Supplemental Figure S4). Significant transcriptional differences of *GhLtps* were found between cultivated upland cotton varieties with longer and shorter fibers. And many *GhLtpXIs* displayed preponderance of expression in longer fibers, which was likely to contribute to the variation of fiber quality (Figures 4, 5). The transcription of several *GhLtpXIs* was verified by qPCR with a higher level or advanced peak in long-fibered cultivar (Figure 5). Furthermore, significant elongation of *Arabidopsis* leaf trichomes induced by overexpression of *GhLtpXIs* suggested functional roles of *GhLtpXIs* in promoting cell elongation (Figure 6).

Since the first genetic map was constructed in cotton, many QTLs for fiber quality traits have been identified (Shen et al., 2005; Wang et al., 2006; Qin et al., 2008; Zhang et al., 2012; Ning et al., 2014; Shao et al., 2014; Shang et al., 2015; Jamshed et al., 2016). After blast with the QTLs for fiber quality traits, *GhLtpI24, GhLtpIV6* and a cluster of *GhLtpXIs* located in chromosome Dt11 (*GhLtpXI13*/*14*/*15*/*17*/*19*/*21*/*23*/*24*/*27*) fell in QTL qFL15.1, qFS-D13-1, and qFM21.1, respectively (Zhang et al., 2012; Ning et al., 2014; Shao et al., 2014). These findings further proved that *GhLtps* especially *GhLtpXIs* are important regulators of fiber development and thus contribute to fiber quality.

LCFA regulates endogenous ethylene biosynthesis in cotton ovules to promote the extensibility of fibers (Qin et al., 2007). The basic function of nsLTPs is to transfer lipids. GhLtps were speculated to bind with various lipids to be responsible for the intracellular and intercellular movement of lipids including fatty acids and thus affect ethylene production and fiber elongation. A GPI-anchored lipid transport protein (GhLTPG1) has been identified to regulate cotton fiber elongation through mediating the transport of phosphatidylinositol monophosphates (Ostergaard et al., 1993; Deng et al., 2016), which supports the speculation. As a consequence of the transportation of lipids, the intercellular fatty acid pools will be regulated by GhLtps, which would cause a feedback regulation on reactions enrolling fatty acids, including LCFA biosynthesis and accumulation. In addition to transferring lipids, nsLTPs can also act as lipid sensors and lipid chaperones (Wong et al., 2017). Plant nsLTPs are supposed to bind a lipid molecular competing with elicitin and mediate signaling (Blein et al., 2002; Maldonado et al., 2002). When nsLTPs present part of a lipid (typically the hydrophilic head group) to another protein, they act as lipid chaperones to mediate signaling (Wang et al., 2005). Thus a hypothesis that GhLtps participant in the signaling of phytohormones involved in fiber development and other signaling pathways mediating fiber development could be proved when functional lipids were isolated as signaling molecules from fibers or the outer integument of ovules.

### *GhLtpXIs* might comprise a possible “fiber clade”

Shortly after the divergence from the same ancestor as *Th. cacao* at least 60 Myr ago (Carvalho et al., 2011), the genus *Gossypium* experienced an abrupt polyploidization with a maternal A-genome propagule resembling *G. herbaceum* and a pollen parent D-genome species resembling *G. raimondii* which diverged ~5–10 Myr ago (Senchina et al., 2003). The nascent AtDt allopolyploid diverged into at least five species, of which two major cultivated species *G. hirsutum* and *G. barbadense* were domesticated independently to spawn textile industry and became a major oilseed. Although *G. hirsutum* has been well domesticated and bred for many cultivated varieties that vary in fiber quality, the evolution of fiber and regulatory mechanism of fiber development are unclarified. Our results suggested that the expanded type XI genes in *G. hirsutum* genome are involved in fiber development. Although GhLtpXIs were phylogenetically close to GhLtpIIs, they evolved to be different in protein structure and MWs. Members of this group contain an extra N-terminal cap (Figure 1). Protein sequences of GhLtpXIs were highly conserved within the ECMs and only varied at the N-terminal (Figure 1). Additionally, the divergence time of *GhLtpXIs* was earlier than other *GhLtps*. It is clear that *GhLtpXIs* form a unique group distinctive from other *GhLtps*.

The sequence of a *G. hirsutum* cultivar reveals many non-reciprocal DNA exchanges between subgenomes that may have contributed to phenotypic innovation that spinnable fiber evolved from the merger of A genome and D genome determining a fibered and a fibreless phenotype, respectively (Paterson et al., 2012). Noticeably, non-reciprocal DNA exchanges were found in cotton type XI genes and most transcribed *GhLtpXIs* in ovules displayed significant expression difference during fiber elongation compared with their orthologs in A or D genome, indicating a correlation between cotton type XI genes and fiber evolution. Therefore the cotton XI genes are speculated to comprise a possible “fiber clade” (Paterson et al., 2012) evolving from polyploidy, non-reciprocal DNA exchanges and duplication events, which would be supported by further sequence comparison of nsLTPs in different cotton species and their evolutional close species and molecular investigation on how these candidates regulate fiber development.

## Author contributions

ZM, CM, and YY designed the experiments. CM and YY conducted the experiments and analyzed the data. ZL, LC, and YZ performed part of the experiments. XL helped in the bioinformatics analyses. LW and GZ helped in growing experimental materials. CM and YY wrote the manuscript. ZM and XW carefully edited the manuscript and analyzed the data. All authors revised the manuscript.

### Conflict of interest statement

The authors declare that the research was conducted in the absence of any commercial or financial relationships that could be construed as a potential conflict of interest.

## Supplementary material

Supplemental Figure S1 Phylogenetic tree of GhLtps. The amino acids of the mature proteins of cotton and Arabidopsis nsLtps were used to build the phylogenetic tree using Maximum likelihood method. The 10 types of nsLTPs and another five AtLtps are showed with different color background. Four clusters are marked against different colors.

Supplemental Figure S2 Gene structure of the *GhLtps*. Exons are represented by green boxes and introns are showed with black lines.

Supplemental Figure S3 Expression profiles of *GhLtpXIs* in different organs and tissues of *G. hirsutum*. Data shown were log_2_-transformed FPKM of each gene which was quantified using RNA-seq data downloaded from CottonFGD. Clustering of expression level is showed on the right. The color bar represents the relative expression level.

Supplemental Figure S4 Expression of *GhLtps* during fiber development. Data shown were log_2_-transformed RPKM. Developmental stages of fiber are indicated in days post anthesis (DPA) above. The color bar represents the relative expression level.

Supplemental Figure S5 Expression trend analysis of *GhLtps*. Number of genes is indicated below the relevant expression pattern.

Supplemental Figure S6 Phylogenetic tree of nsLTPs in cotton species. The full lengths of mature protein sequences were used to construct the phylogenetic tree using a Neighbor-Joining method. The same color was used to present orthologous gene pairs and non-reciprocal DNA exchanges were labeled with different dots.

Supplemental Table S1 List of primers used in this study.

Supplemental Table S2 Diversity of the eight cysteine motif.

Supplemental Table S3 Classification of nsLTP genes in cotton, *Arabidopsis*, rape, rice, cacao and grape.

Supplemental Table S4 Ka/Ks analysis for the duplicated gene pairs.

## References

[B1] Basra A. S. Malik C. P. (1984). Development of the cotton fiber. Int. Rev. Cytol. 89, 65–113.

[B2] Blein J. P. Coutos-Thévenot P. Marion D. Ponchet M. (2002). From elicitins to lipid-transfer proteins: a new insight in cell signalling involved in plant defence mechanisms. Trends in Plant Sci. 7, 293–296. 10.1016/S1360-1385(02)02284-7 12119165

[B3] Boutrot F. Chantret N. Gautier M. F. (2008). Genome-wide analysis of the rice and Arabidopsis non-specific lipid transfer protein (nsLTP) gene families and identification of wheat nsLtp genes by EST data mining. BMC Genomics 9:86. 10.1186/1471-2164-9-86 18291034 PMC2277411

[B4] Carvalho A. O. Gomes V. M. (2007). Role of plant lipid transfer proteins in plant cell physiology-a concise review. Peptides 28, 1144–1153. 10.1016/j.peptides.2007.03.004 17418913

[B5] Carvalho M. R. Herrera F. A. Jaramillo C. A. Wing S. L. Callejas R. (2011). Paleocene malvaceae from northern south America and their biogeographical implications. Am. J. Bot. 98, 1337–1355. 10.3732/ajb.1000539 21821594

[B6] Chae K. Gonong B. J. Kim S. C. Kieslich C. A. Morikis D. Balasubramanian S.. (2010). A multifaceted study of stigma/style cysteine-rich adhesin (SCA)-like Arabidopsis lipid transfer proteins (LTPs) suggests diversified roles for these LTPs in plant growth and reproduction. J. Exp. Bot. 61, 4277–4290. 10.1093/jxb/erq228 20667964 PMC2955742

[B7] Charvolin D. Douliez J. P. Marion D. Cohenaddad C. Pebaypeyroula E. (1999). The crystal structure of a wheat non-specific lipid transfer protein (ns-LTP1) complexed with two molecules of phospholipid at 2.1 A resolution. Febs J. 264, 562–568. 10.1046/j.1432-1327.1999.00667.x 10491104

[B8] Chen C. Chen G. Hao X. Cao B. Chen Q. Liu S.. (2011). CaMF2, an anther-specific lipid transfer protein (LTP) gene, affects pollen development in *Capsicum annuum* L. Plant Sci. 181, 439–448. 10.1016/j.plantsci.2011.07.003 21889050

[B9] Cheng H. C. Cheng P. T. Peng P. Lyu P. C. Sun Y. J. (2004). Lipid binding in rice non-specific lipid transfer protein-1 complexes from *Oryza sativa*. Protein. Sci. 13:2304. 10.1110/ps.04799704 15295114 PMC2280015

[B10] Deng T. Yao H. Y. Wang J. Wang J. Xue H. W. Zuo K. J. (2016). GhLTPG1, a cotton GPI-anchored lipid transfer protein, regulates the transport of phosphatidylinositol monophosphates and cotton fiber elongation. Sci. Rep. 6:26829. 10.1038/srep26829 27311358 PMC4911556

[B11] Douliez J. P. Michon T. Elmorjani K. Marion D. (2000). Structure, biological and technological functions of lipid transfer proteins and indolines, the major lipid binding proteins from cereal kernels. J. Cer. Sci. 32, 1–20. 10.1006/jcrs.2000.0315

[B12] Edstam M. M. Blomqvist K. Eklof A. Wennergren U. Edqvist J. (2013). Co-expression patterns indicate that GPI-anchored non-specific lipid transfer proteins are involved in accumulation of cuticular wax, suberin and sporopollenin. Plant Mol. Biol. 83, 625–649. 10.1007/s11103-013-0113-5 23893219

[B13] Edstam M. M. Edqvist J. (2014). Involvement of GPI-anchored lipid transfer proteins in the development of seed coats and pollen in *Arabidopsis thaliana*. Physiol. Plant. 152, 32–42. 10.1111/ppl.12156 24460633

[B14] Guan X. Y. Li Q. J. Shan C. M. Wang S. Mao Y. B. Wang L. J.. (2008). The HD-Zip IV gene *GaHOX1* from cotton is a functional homologue of the *Arabidopsis* GLABRA2. Physiol. Plant. 134, 174–182. 10.1111/j.1399-3054.2008.01115.x 18507789

[B15] Guan X. Y. Pang M. X. Nah G. Shi X. L. Ye W. X. David M. S.. (2014). miR828 and miR858 regulate homoeologous MYB2 gene functions in *Arabidopsis* trichome and cotton fibre development. Nat. Commun. 5:3050. 10.1038/ncomms4050 24430011

[B16] Han G. W. Lee J. Y. Song H. K. Chang C. Min K. Moon J.. (2001). Structural basis of non-specific lipid binding in maize lipid-transfer protein complexes revealed by high-resolution X-ray crystallography. J. Mol. Biol. 308, 263–278. 10.1006/jmbi.2001.4559 11327766

[B17] Han H. C. Huang Y. Q. Wang J. Zuo K. J. (2013). Cloning and expression pattern analysis of *Gossypium hirsutum* lipid transfer protein gene family. J. Agr. Sci. Tech. 15, 84–90.

[B18] Han J. Pan Y. X. Wang X. F. Zhang Y. Ma Z. Y. (2016). Antisense expression of *Gossypium barbadense UGD6* in *Arabidopsis thaliana* significantly alters cell wall composition. Sci. China Life Sci. 59, 213–218. 10.1007/s11427-016-5004-y 26810898

[B19] Hsu C.-Y. Jenkins J. N. Saha S. Ma D.-P. (2005). Transcriptional regulation of the lipid transfer protein gene *LTP3* in cotton fibers by a novel MYB protein. Plant Sci. 168, 167–181. 10.1016/j.plantsci.2004.07.033

[B20] Huang Y. Q. Liu X. Tang K. X. Zuo K. J. (2013). Functional analysis of the seed coat-specific gene GbMYB2 from cotton. Plant Physiol. Bioch. 73, 16–22. 10.1016/j.plaphy.2013.08.004 24036393

[B21] Jamshed M. Jia F. Gong J. W. Palanga K. K. Shi Y. Z. Li J. W.. (2016). Identification of stable quantitative trait loci (QTLs) for fiber quality traits across multiple environments in *Gossypium hirsutum* recombinant inbred line population. BMC Genomics 17:197. 10.1186/s12864-016-2560-2 26951621 PMC4782318

[B22] Ji S. J. Lu Y. C. Feng J. X. Wei G. Li J. Shi Y. H.. (2003). Isolation and analyses of genes preferentially expressed during early cotton fiber development by subtractive PCR and cDNA array. Nucl. Acids Res. 31, 2534–2543. 10.1093/nar/gkg358 12736302 PMC156040

[B23] Joshi P. C. Wadhwani A. M. Johri B. M. (1967). Morphological and embryological studies of *Gossypium*, L. Indian J. Agr. Res. 33, 37–93.

[B24] Kader J. C. (1996). Lipid-transfer proteins in plants. Ann. Plant Physiol. Plant Mol. Biol. 47, 627–654. 15012303 10.1146/annurev.arplant.47.1.627

[B25] Kim H. J. Triplett B. A. (2001). Cotton fiber growth in planta and *in vitro* models for plant cell elongation and cell wall biogenesis. Plant Physiol. 127, 1361–1366. 10.1104/pp.010724 11743074 PMC1540163

[B26] Kong X. P. Lv W. Jiang S. S. Zhang D. Cai G. H. Pan J. W.. (2013). Genome-wide identification and expression analysis of calcium-dependent protein kinase in maize. BMC Genomics 14:433. 10.1186/1471-2164-14-433 23815483 PMC3704972

[B27] Kragelund B. B. Bech L. M. Poulsen F. M. (1997). Barley lipid-transfer protein complexed with palmitoyl CoA: the structure reveals a hydrophobic binding site that can expand to fit both large and small lipid-like ligands. Structure 5, 291–306. 9032083 10.1016/s0969-2126(97)00186-x

[B28] Kumar S. Kumar K. Pandey P. Rajamani V. Padmalatha K. V. Dhandapani G.. (2013). Glycoproteome of elongating cotton fiber cells. Mol. Cell. Pro. 12, 3677–3689. 10.1074/mcp.M113.030726 24019148 PMC3861716

[B29] Lee S. B. Go Y. S. Bae H. J. Park J. H. Cho S. H. Cho H. J.. (2009). Disruption of glycosylphosphatidylinositol-anchored lipid transfer protein gene altered cuticular lipid composition, increased plastoglobules, and enhanced susceptibility to infection by the fungal pathogen alternaria brassicicola. Plant Physiol. 150, 42–54. 10.1104/pp.109.137745 19321705 PMC2675750

[B30] Lei L. Chen L. Shi X. F. Li Y. X. Wang J. Y. Chen D. S.. (2014). A nodule-specific lipid transfer protein asE246 participates in transport of plant-synthesized lipids to symbiosome membrane and is essential for nodule organogenesis in chinese milk vetch. Plant Physiol. 164, 1045–1058. 10.1104/pp.113.232637 24367021 PMC3912078

[B31] Li F. G. Fan G. Y. Lu C. R. Xiao G. H. Zou C. S. Kohel R. J.. (2015). Genome sequence of cultivated Upland cotton (*Gossypium hirsutum* TM-1) provides insights into genome evolution. Nat. Biotech. 33, 524–530. 10.1038/nbt.3208 25893780

[B32] Li J. Gao G. Z. Xu K. Chen B. Y. Yan G. X. Li F.. (2014). Genome-wide survey and expression analysis of the putative non-specific lipid transfer proteins in *Brassica rapa* L. PLoS ONE 9:e84556. 10.1371/journal.pone.0084556 24497919 PMC3908880

[B33] Liu B. L. Zhu Y. C. Zhang T. T. (2015). The R3-MYB gene *GhCPC* negatively regulates cotton fiber elongation. PLoS ONE 10:e0116272. 10.1145/2818302 25646816 PMC4315419

[B34] Liu H. C. Creech R. G. Jenkins J. N. Ma D. P. (2000). Cloning and promoter analysis of the cotton lipid transfer protein gene Ltp3. Bioch. Bioph. Acta 1487, 106–111. 10.1016/S1388-1981(00)00072-X 11004611

[B35] Liu W. Li W. He Q. Daud M. K. Chen J. Zhu S. J. (2014). Genome-wide survey and expression analysis of calcium-dependent protein kinase in *Gossypium raimondii*. PLoS ONE 9:e98189. 10.1371/journal.pone.0098189 24887436 PMC4041719

[B36] Ma D. P. Liu H. C. Tan H. P. Creech R. G. Jenkins J. N. Chang Y. F. (1997). Cloning and characterization of a cotton lipid transfer protein gene specifically expressed in fiber cells. Bioch. Bioph. Acta 1344, 111–114. 10.1016/S0005-2760(96)00166-X 9030188

[B37] Ma D. P. Tan H. Si Y. Creech R. G. Jenkins J. N. (1995). Differential expression of a lipid transfer protein gene in cotton fiber. Bioch. Bioph. Acta 1257, 81–84. 7599183 10.1016/0005-2760(95)00077-p

[B38] Ma Z. Y. He S. P. Wang X. F. Sun J. L. Zhang Y. Zhang G. Y.. (2018). Resequencing a core collection of upland cotton identifies genomic variation and loci influencing fiber quality and yield. Nat. Genet. 50, 803–813. 10.1038/s41588-018-0119-7 29736016

[B39] Machado A. Wu Y. R. Yang Y. M. Llewellyn D. J. Dennis E. S. (2009). The MYB transcription factor GhMYB25 regulates early fibre and trichome development. Plant J. 59, 52–62. 10.1111/j.1365-313X.2009.03847.x 19309462

[B40] Maere S. De Bodt S. Raes J. Casneuf T. Montagu M. V. Kuiper M. (2005). Modeling gene and genome duplications in eukaryotes. Proc. Nat. Acad. Sci. U.S.A. 102, 5454–5459. 10.1073/pnas.0501102102 15800040 PMC556253

[B41] Maldonado A. M. Doerner P. Dixon R. A. Lamb C. J. Cameron R. K. (2002). A putative lipid transfer protein involved in systemic resistance signalling in *Arabidopsis*. Nature 419, 399–403. 10.1038/nature00962 12353036

[B42] Molina A. Segura A. Garcia-Olmedo F. (1993). Lipid transfer proteins (nsLTPs) from barley and maize leaves are potent inhibitors of bacterial and fungal plant pathogens. Febs Letters 316, 119–122. 8420795 10.1016/0014-5793(93)81198-9

[B43] Ning Z. Y. Chen H. Mei H. X. Zhang T. Z. (2014). Molecular tagging of QTLs for fiber quality and yield in the upland cotton cultivar Acala-Prema. Euphytica 195, 143–156. 10.1007/s10681-013-0990-3

[B44] Orford S. J. Timmis J. N. (2000). Expression of a lipid transfer protein gene family during cotton fibre development. Bioch. Bioph. Acta 1483, 275–284. 10.1016/s1388-1981(99)00194-8 10634943

[B45] Ostergaard J. Vergnolle C. Schoentgen F. Kader J. C. (1993). Acyl-binding/lipid-transfer proteins from rape seedlings, a novel category of proteins interacting with lipids. Bioch. Bioph. Acta 1170, 109–117. 10.1016/0005-2760(93)90059-i 8399333

[B46] Paterson A. H. Wendel J. F. Gundlach H. Guo H. Jenkins J. Jin D. C.. (2012). Repeated polyploidization of *Gossypium* genomes and the evolution of spinnable cotton fibres. Nature 492, 423–427. 10.1038/nature11798 23257886

[B47] Pu L. Li Q. Fan X. P. Yang W. C. Xue Y. B. (2008). The R2R3 MYB transcription factor GhMYB109 is required for cotton fiber development. Genetics 180, 811–820. 10.1534/genetics.108.093070 18780729 PMC2567382

[B48] Pyee J. Yu H. S. Kolattukudy P. E. (1994). Identification of a lipid transfer protein as the major protein in the surface wax of broccoli (*Brassica oleracea*) leaves. Arch. Biol. Biol. 311, 460–468. 10.1006/abbi.1994.1263 8203911

[B49] Qin H. D. Guo W. Z. Zhang Y. M. Zhang T. Z. (2008). QTL mapping of yield and fiber traits based on a four-way cross population in *Gossypium hirsutum* L. Theor. Appl. Genet. 117:883. 10.1007/s00122-008-0828-x 18604518

[B50] Qin Y. M. Hu C. Y. Pang Y. Kastaniotis A. J. Hiltunen J. K. Zhu Y. X. (2007). Saturated very-long-chain fatty acids promote cotton fiber and Arabidopsis cell elongation by activating ethylene biosynthesis. Plant Cell 19, 3692–3704. 10.1105/tpc.107.054437 17993622 PMC2174872

[B51] Qin Y. M. Zhu Y. X. (2011). How cotton fibers elongate: a tale of linear cell-growth mode. Curr. Opin. Plant Biol. 14, 106–111. 10.1016/j.pbi.2010.09.010 20943428

[B52] Sarowar S. J. Kim Y. J. Kim K. D. Hwang B. K. Ok S. H. Shin J. S. (2009). Overexpression of lipid transfer protein (LTP) genes enhances resistance to plant pathogens and LTP functions in long-distance systemic signaling in tobacco. Plant Cell Rep. 28, 419–427. 10.1007/s00299-008-0653-3 19089429

[B53] Schweiger W. Steiner B. Ametz C. Siegwart G. Wiesenberger G. Berthiller F.. (2013). Transcriptomic characterization of two major Fusarium resistance quantitative trait loci (QTLs), Fhb1 and Qfhs.ifa-5A, identifies novel candidate genes. Mol. Plant Pathol. 14, 772–785. 10.1111/mpp.12048 23738863 PMC3902993

[B54] Senchina D. S. Alvarez I. Cronn R. C. Liu B. Rong J. K. Noves R. D.. (2003). Rate variation among nuclear genes and the age of polyploidy in *Gossypium*. Mol. Biol. Evol. 20, 633–643. 10.1093/molbev/msg065 12679546

[B55] Shang L. G. Liang Q. Z. Wang Y. M. Wang X. C. Wang K. B. Abduweli A. (2015). Identification of stable QTLs controlling fiber traits properties in multi-environment using recombinant inbred lines in Upland cotton (*Gossypium hirsutum* L.). Euphytica 205, 877–888. 10.1007/s10681-015-1434-z

[B56] Shangguan X. X. Yang C. Q. Zhang X. F. Wang L. J. (2016). Functional characterization of a basic helix-loop-helix (bHLH) transcription factor GhDEL65 from cotton (*Gossypium hirsutum*). Physiol. Plant. 158, 200–212. 10.1111/ppl.12450 27080593

[B57] Shao Q. S. Zhang F. J. Tang S. Y. Liu Y. Fang X. M. Liu D. X. (2014). Identifying QTL for fiber quality traits with three upland cotton (*Gossypium hirsutum* L.) populations. Euphytica 198, 43–58. 10.1007/s10681-014-1082-8

[B58] Shen X. L. Guo W. Z. Zhu X. F. Yuan Y. L. Yu J. Z. Kohel R. J. (2005). Molecular mapping of QTLs for fiber qualities in three diverse lines in Upland cotton using SSR markers. Mol. Breed. 15, 169–181. 10.1007/s11032-004-4731-0

[B59] Shin H. S. Bleecker A. B. (2003). Expansion of the receptor-like kinase/pelle gene family and receptor-like proteins in *Arabidopsis*. Plant Physiol. 132, 530–543. 10.1104/pp.103.021964 12805585 PMC166995

[B60] Sodano P. Caille A. Sy D. De P. G. Marion D. Ptak M. (1997). 1H NMR and fluorescence studies of the complexation of DMPG by wheat non-specific lipid transfer protein. Global fold of the complex. Febs Lett. 416, 130–134. 9369197 10.1016/s0014-5793(97)01185-x

[B61] Stewart J. M. D. (1975). Fiber initiation on the cotton ovule (*Gossypium Hirsutum*). Am. J. Bot. 62, 723–730.

[B62] Walford S. A. Wu Y. R. Llewellyn D. J. Dennis E. S. (2011). GhMYB25-like: a key factor in early cotton fibre development. Plant J. 65, 785–797. 10.1111/j.1365-313X.2010.04464.x 21235650

[B63] Wan S. W. Welti R. Moreau R. A. Chapman K. D. (2005). Identification and quantification of glycerolipids in cotton fibers: reconciliation with metabolic pathway predictions from DNA databases. Lipids 40, 773–785. 10.1007/s11745-005-1439-4 16296396

[B64] Wang B. H. Guo W. Z. Zhu X. F. Wu Y. T. Huang N. T. Zhang T. (2006). QTL mapping of fiber quality in an elite hybrid derived-RIL population of upland cotton. Euphytica 152, 367–378. 10.1007/s10681-006-9224-2

[B65] Wang G. Zhao G. H. Jia Y. H. Du X. M. (2013). Identification and characterization of cotton genes involved in fuzz-fiber development. J. Integr. Plant Biol. 55, 619–630. 10.1111/jipb.12072 23710824

[B66] Wang P. Y. Weng J. Anderson R. G. (2005). OSBP is a cholesterol-regulated scaffolding protein in control of ERK 1/2 activation. Science 307, 1472–1476. 10.1126/science.1107710 15746430

[B67] Wang Y. P. Wang X. Y. Tang H. B. Tan X. Ficklin S. P. Feltus F. A.. (2011). Modes of gene duplication contribute differently to genetic novelty and redundancy, but show parallels across divergent angiosperms. PLoS ONE 6:e28150. 10.1371/journal.pone.0028150 22164235 PMC3229532

[B68] Wei H. L. Li W. Sun X. W. Zhu S. J. Zhu J. (2013). Systematic analysis and comparison of nucleotide-binding site disease resistance genes in a diploid cotton *Gossypium raimondii*. PLoS ONE 8:e68435. 10.1371/journal.pone.0068435 23936305 PMC3735570

[B69] Wei K. Zhong X. (2014). Non-specific lipid transfer proteins in maize. BMC Plant Biol. 14:281. 10.1186/s12870-014-0281-8 25348423 PMC4226865

[B70] Wong L. H. Čopič A. K. Levine T. P. (2017). Advances on the transfer of lipids by lipid transfer proteins. Trends Biochem. Sci. 42, 516–530. 10.1016/j.tibs.2017.05.001 28579073 PMC5486777

[B71] Yu H. Ito T. Wellmer F. Meyerowitz E. M. (2004). Repression of AGAMOUS-LIKE 24 is a crucial step in promoting flower development. Nat. Genet. 36, 157–161. 10.1038/ng1286 14716314

[B72] Zhang D. S. Liang W. Q. Yin C. S. Zong J. Gu F. W. Zhang D. B. (2010). *OsC6*, encoding a lipid transfer protein, is required for postmeiotic anther development in rice. Plant Physiol. 154, 149–162. 10.1104/pp.110.158865 20610705 PMC2938136

[B73] Zhang K. Zhang J. Ma J. Tang S. Y. Liu D. J. Teng Z. H. (2012). Genetic mapping and quantitative trait locus analysis of fiber quality traits using a three-parent composite population in upland cotton (*Gossypium hirsutum* L.). Mol. Breed. 29, 335–348. 10.1007/s11032-011-9549-y

[B74] Zhang T. Z. Hu Y. Jiang W. K. Guan X. Y. Chen J. D. Zhang J. Z.. (2015). Sequencing of allotetraploid cotton (*Gossypium hirsutum* L. acc. TM-1) provides a resource for fiber improvement. Nat. Biotech. 33, 531–537. 10.1038/nbt.3207 25893781

[B75] Zhang Z. Y. Chao M. N. Wang S. F. Bu J. J. Tang J. X. Li F.. (2016). Proteome quantification of cotton xylem sap suggests the mechanisms of potassium-deficiency-induced changes in plant resistance to environmental stresses. Sci. Rep. 6:21060. 10.1038/Srep21060 26879005 PMC4754703

