# Supplementary figures and images for: Systematic Analysis of Cotton Non-specific Lipid Transfer Protein Family Revealed a Special Group That Is Involved in Fiber Elongation
Source: Front Plant Sci. 2018 Sep 19;9:1285. doi: 10.3389/fpls.2018.01285 (PMC6156462; doi:10.3389/fpls.2018.01285)

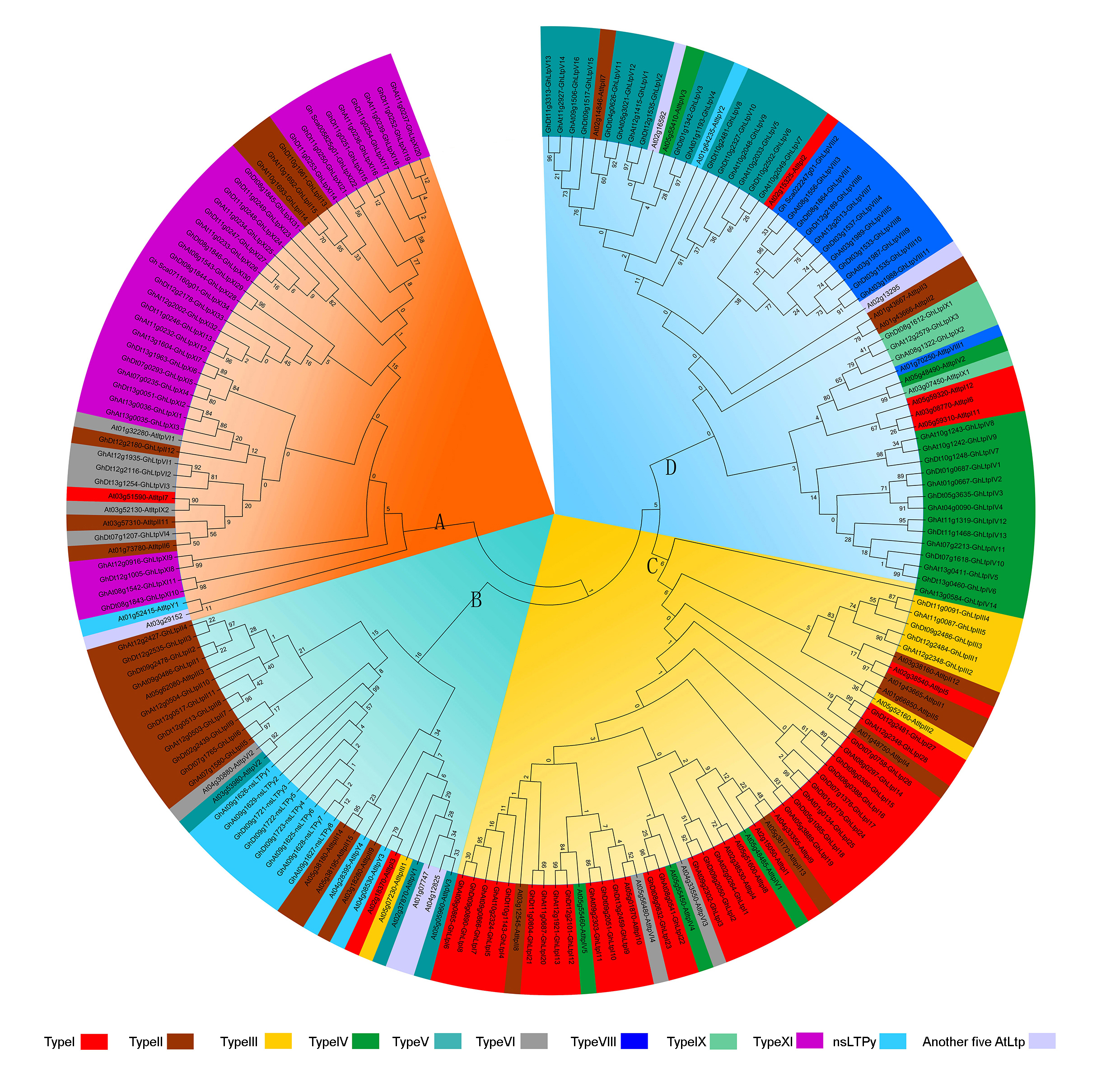

Supplement: Supplemental Figure S1 — Phylogenetic tree of GhLtps. The amino acids of the mature proteins of cotton and Arabidopsis nsLtps were used to build the phylogenetic tree using Maximum likelihood method. The 10 types of nsLTPs and another five AtLtps are showed with different color background. Four clusters are marked against different colors. [file Image_1.jpg]

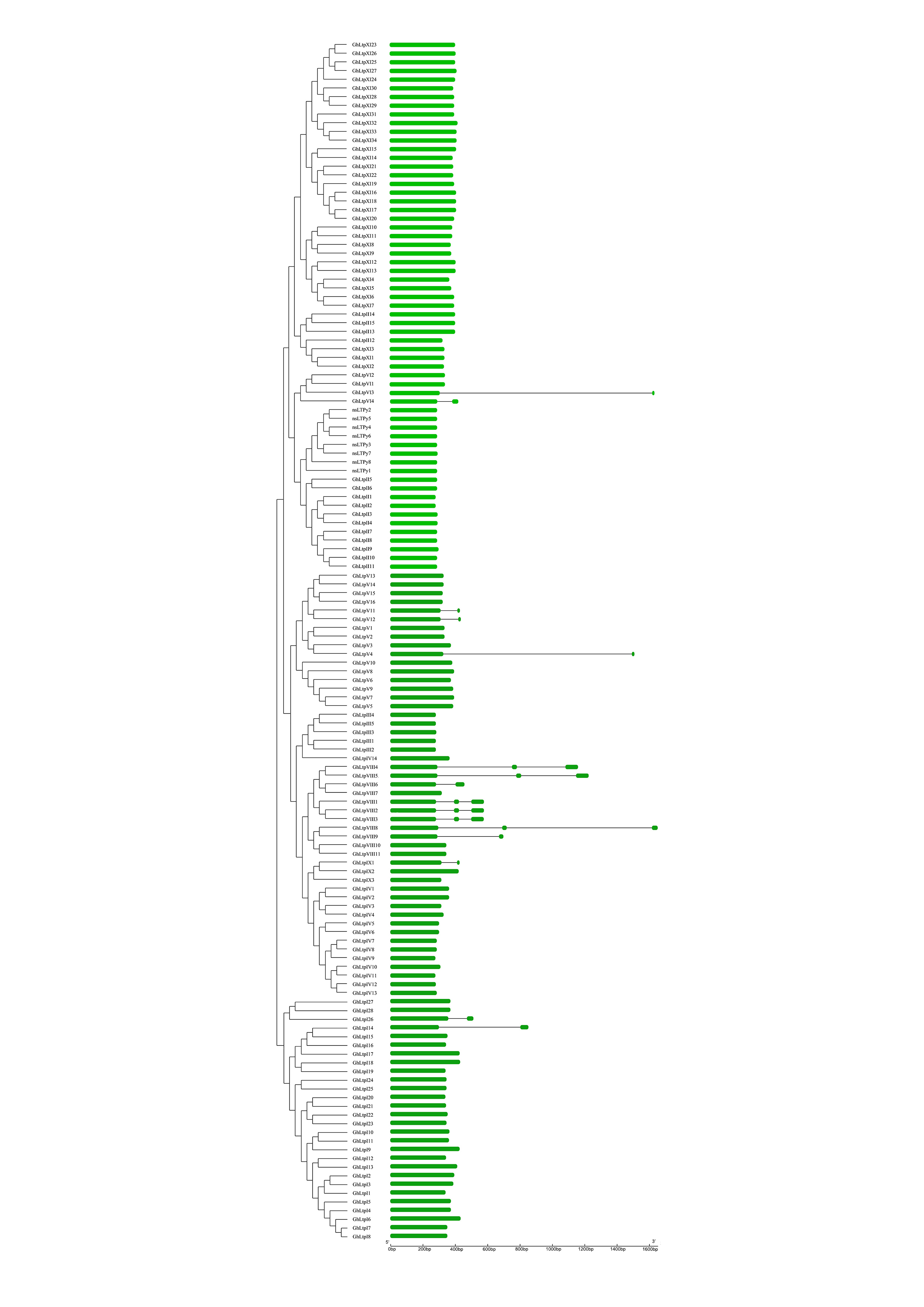

Supplement: Supplemental Figure S2 — Gene structure of the GhLtps. Exons are represented by green boxes and introns are showed with black lines. [file Image_2.JPEG]

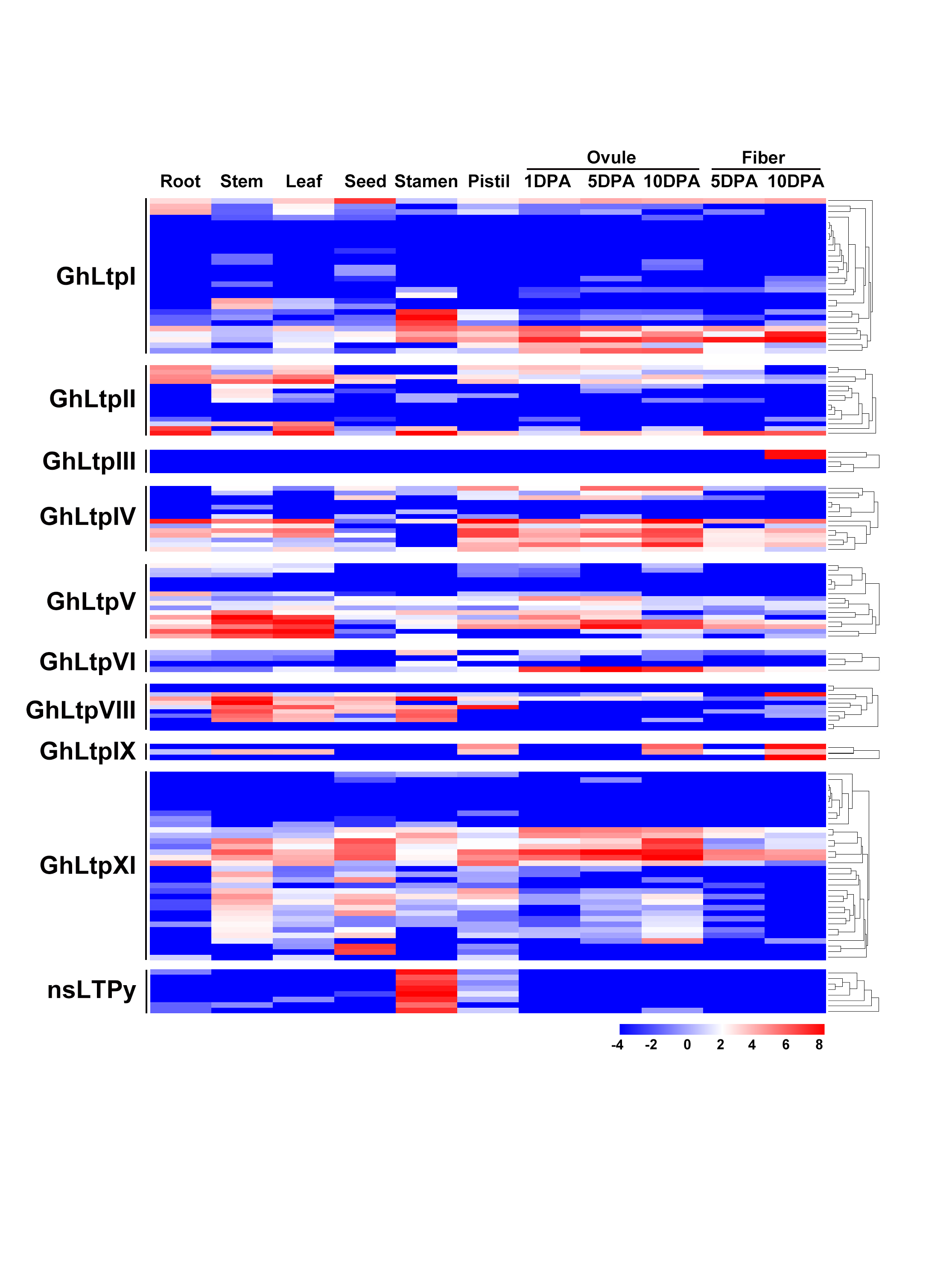

Supplement: Supplemental Figure S3 — Expression profiles of GhLtpXIs in different organs and tissues of G. hirsutum. Data shown were log2-transformed FPKM of each gene which was quantified using RNA-seq data downloaded from CottonFGD. Clustering of expression level is showed on the right. The color bar represents the relative expression level. [file Image_3.JPEG]

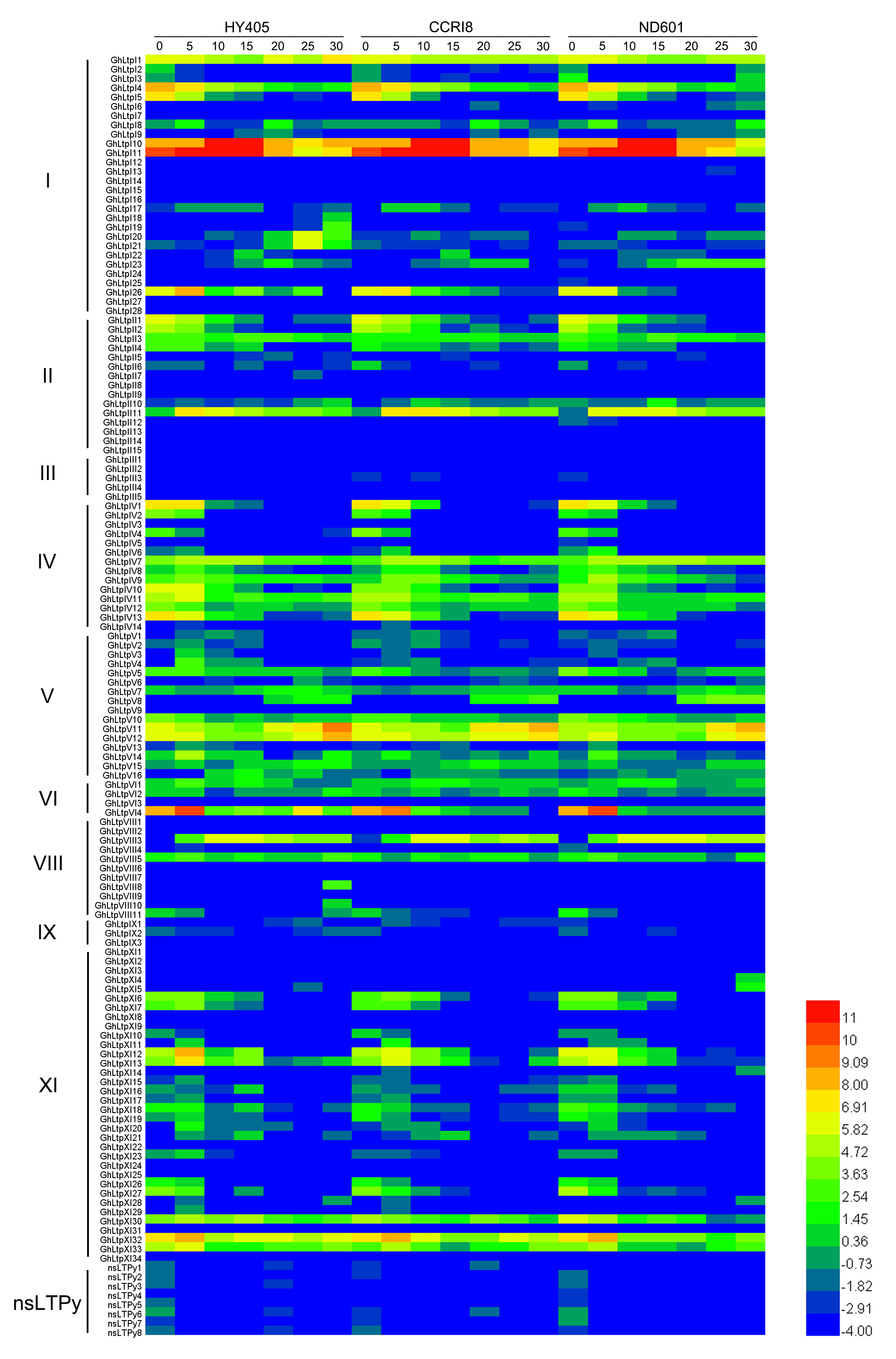

Supplement: Supplemental Figure S4 — Expression of GhLtps during fiber development. Data shown were log2-transformed RPKM. Developmental stages of fiber are indicated in days post anthesis (DPA) above. The color bar represents the relative expression level. [file Image_4.JPEG]

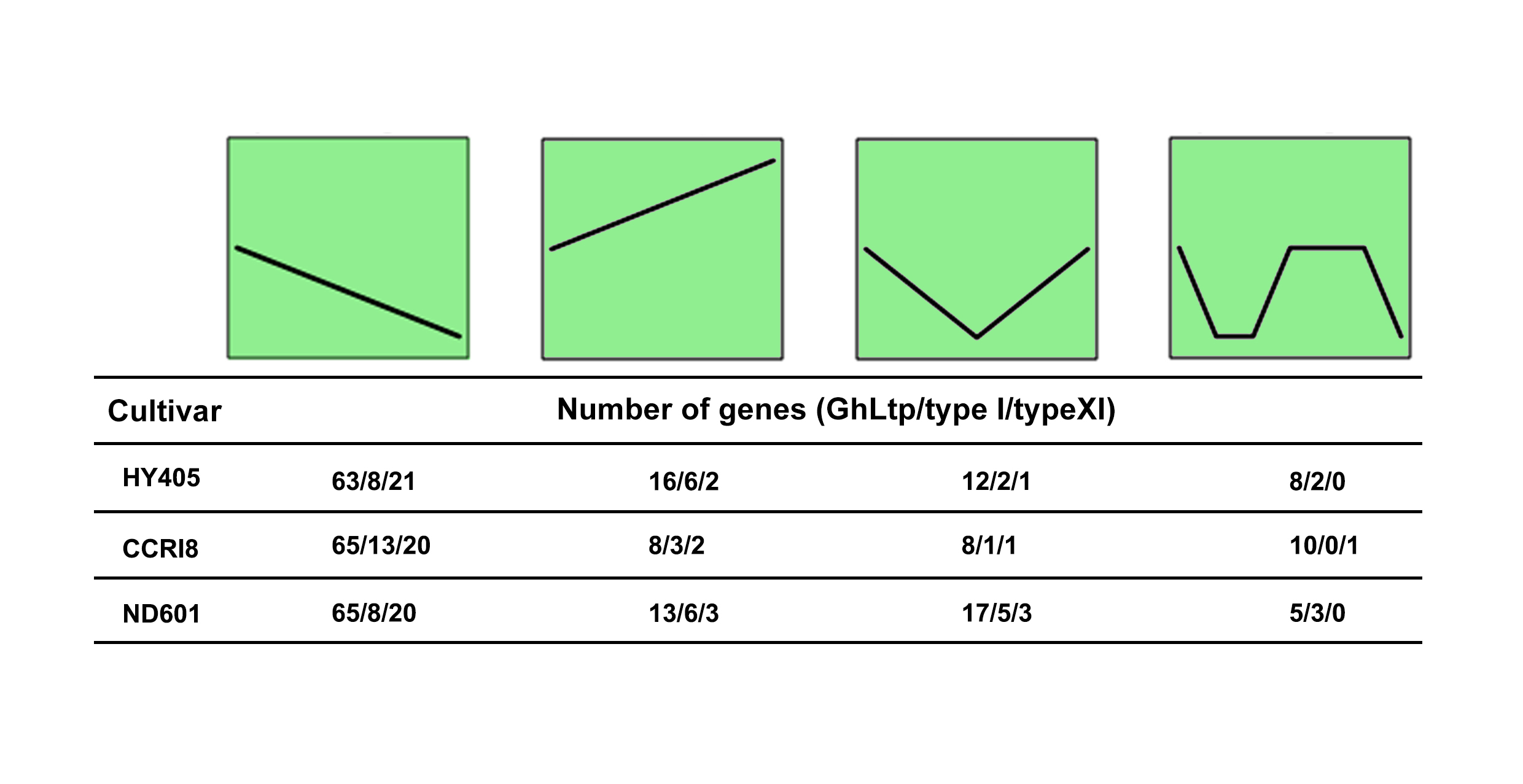

Supplement: Supplemental Figure S5 — Expression trend analysis of GhLtps. Number of genes is indicated below the relevant expression pattern. [file Image_5.JPEG]

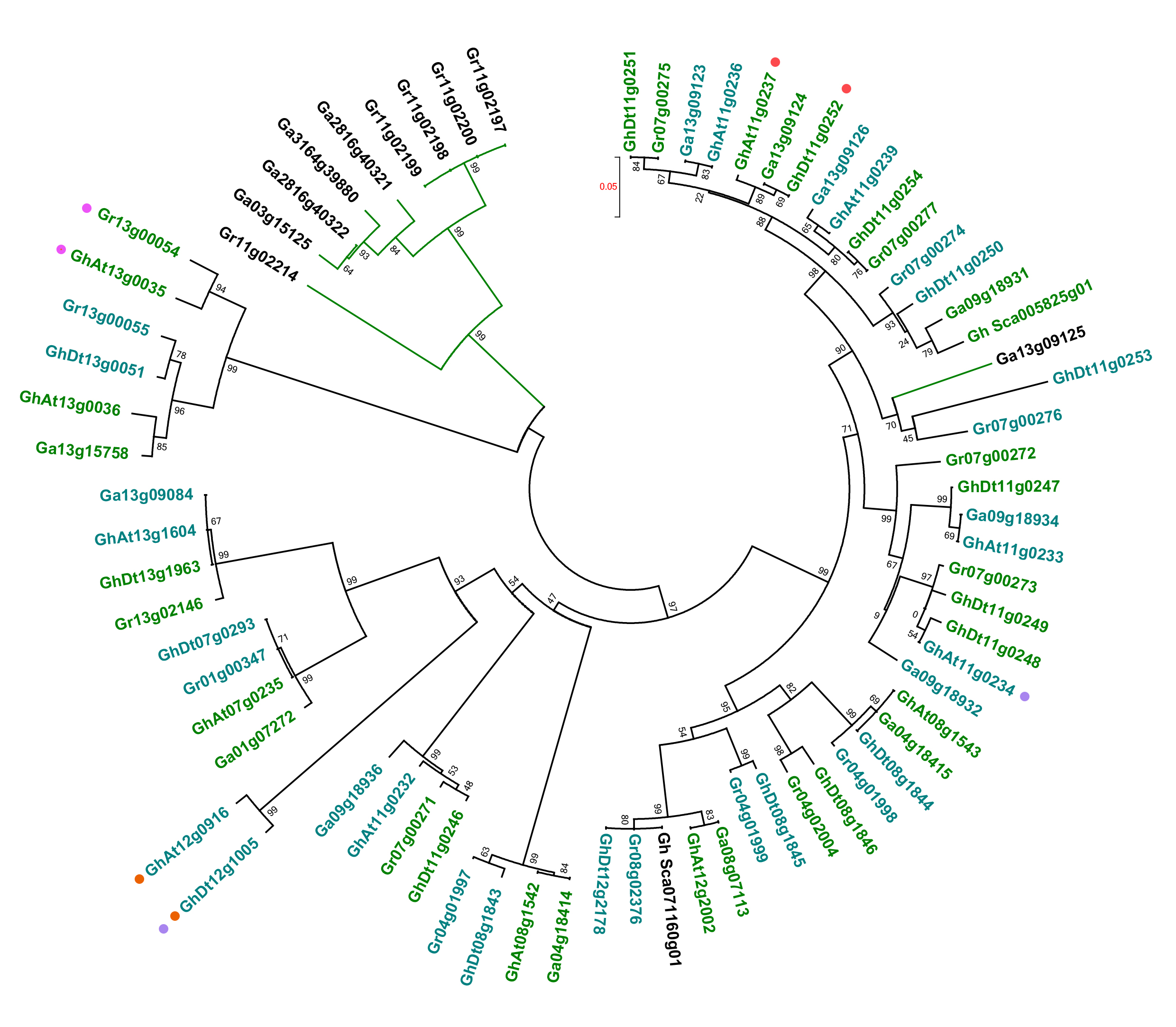

Supplement: Supplemental Figure S6 — Phylogenetic tree of nsLTPs in cotton species. The full lengths of mature protein sequences were used to construct the phylogenetic tree using a Neighbor-Joining method. The same color was used to present orthologous gene pairs and non-reciprocal DNA exchanges were labeled with different dots. [file Image_6.JPEG]
